# Supplementary material for: mHealth interventions for postpartum family planning in LMICs: A realist review
Source: PLOS Glob Public Health. 2024 Jul 18;4(7):e0003432. doi: 10.1371/journal.pgph.0003432 (PMC11257288; doi:10.1371/journal.pgph.0003432)
Supplement: S1 Text — (DOCX) [file pgph.0003432.s001.docx]

## mMitra Program

### Development

mMitra is an mHealth programme that delivers automated voice-messages to pregnant and postpartum women with the aim of improving antenatal and postnatal health knowledge in low-income women across India (1). mMitra was modelled after the Mobile Alliance for Maternal Action (MAMA) intervention from South Africa and has been implemented by ARMMAN, a non-profit that develops and implements digital solutions to ‘**a**dvance the **r**eduction of **m**aternal **m**ortality and morbidity of mothers **a**nd **n**eonates’ (ARMMAN) in India, since 2014 (2). Although initially piloted in Mumbai’s slum communities, mMitra is currently available in nine Indian states including Telangana, Madhya Pradesh, and Odisha and has reached approximately 2.5 million women (1). The MAMA intervention, and mMitra, is underpinned by the assumption that access to easily understandable and engaging information aligning with women’s antenatal and postnatal stage will encourage them to perform necessary self-care measures and ‘seek needed health services’ (3). The MAMA intervention’s theory of change (TOC) and priority outcomes are presented in *Figure I.*


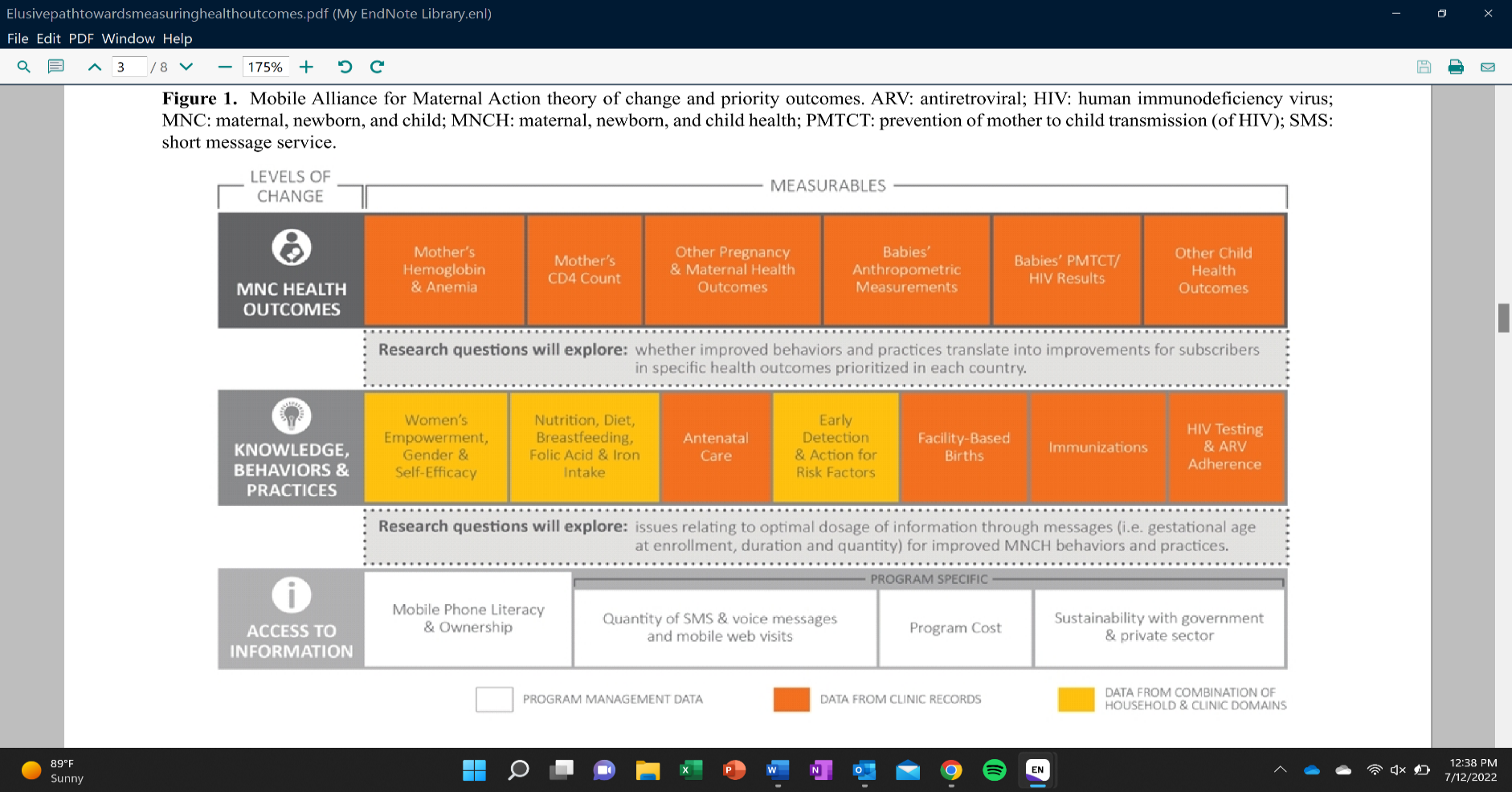


MAMA Theory of change and priority outcomes which was applied to the development of mMitra.

### Programme content and recruitment

The mMitra programme consists of 141, culturally tailored audio messages (2) developed by the BabyCenter (4) using WHO and Indian National Health Mission Guidelines on self-care during pregnancy and infant development (5). mMitra voice-messages were initially developed in English and subsequently translated into Hindi and Marathi and tested for cultural appropriateness prior to launching (1). At registration, subscribers select a preferred call timeslot and language (Hindi or Marathi). Women are enrolled into the mMitra programme at two types of recruitment sites (2):

1. Community health workers known as *Sakhis* are posted in labour and delivery wards of both government and private hospitals to enrol women attending their first pregnancy-related check-up.
2. *Sakhis* enrol women in slum communities through partner NGOs.

Women who enrol in the mMitra programme have opportunities to opt-out of the service. The full mMitra enrolment process is detailed in *Figure II* on page 13. The mMitra programme is stage-based; every subscriber receives content corresponding to their stage of pregnancy or infant development (2). A summary of mMitra’s content is provided in *Appendix I.*


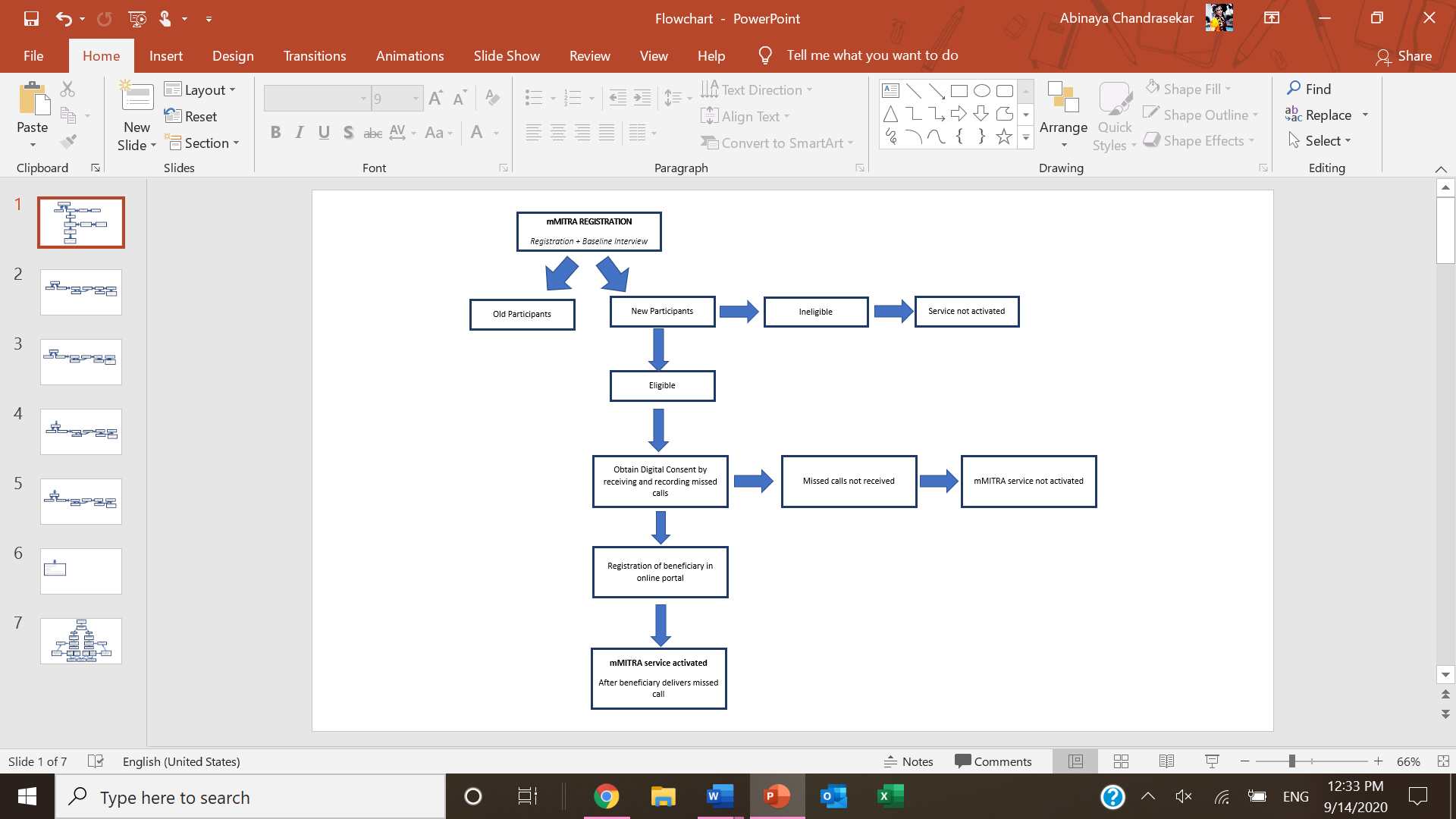

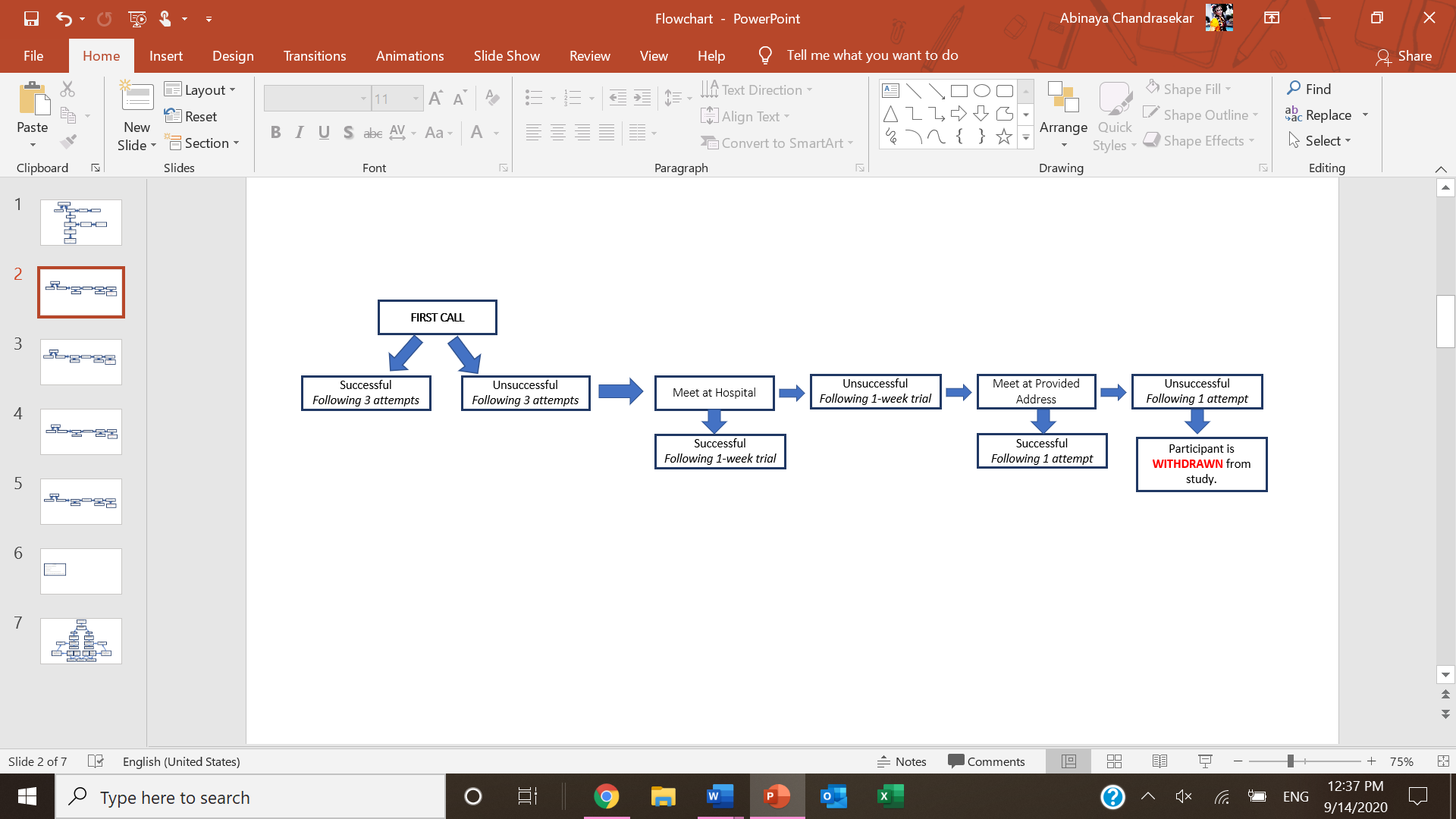

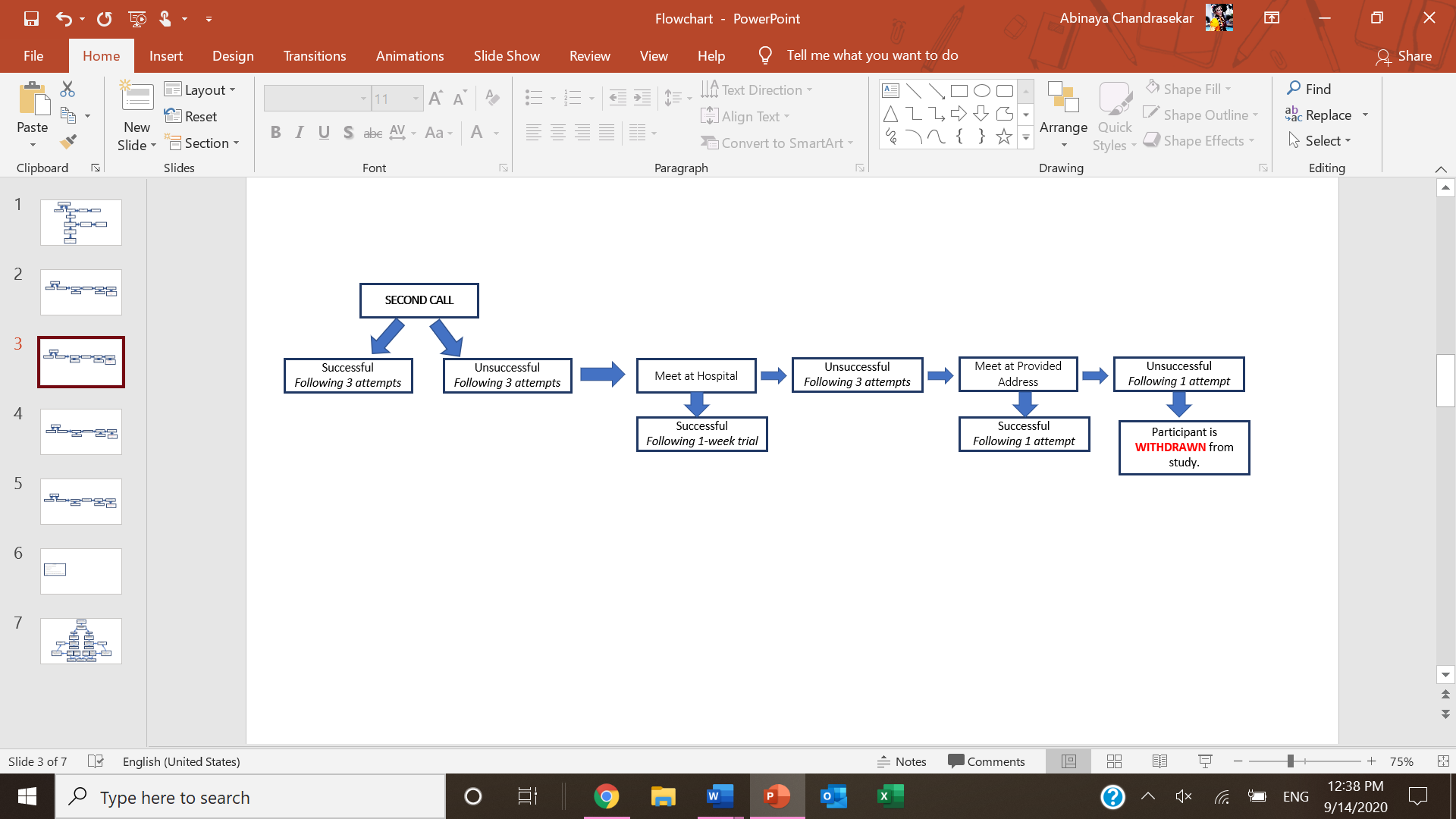

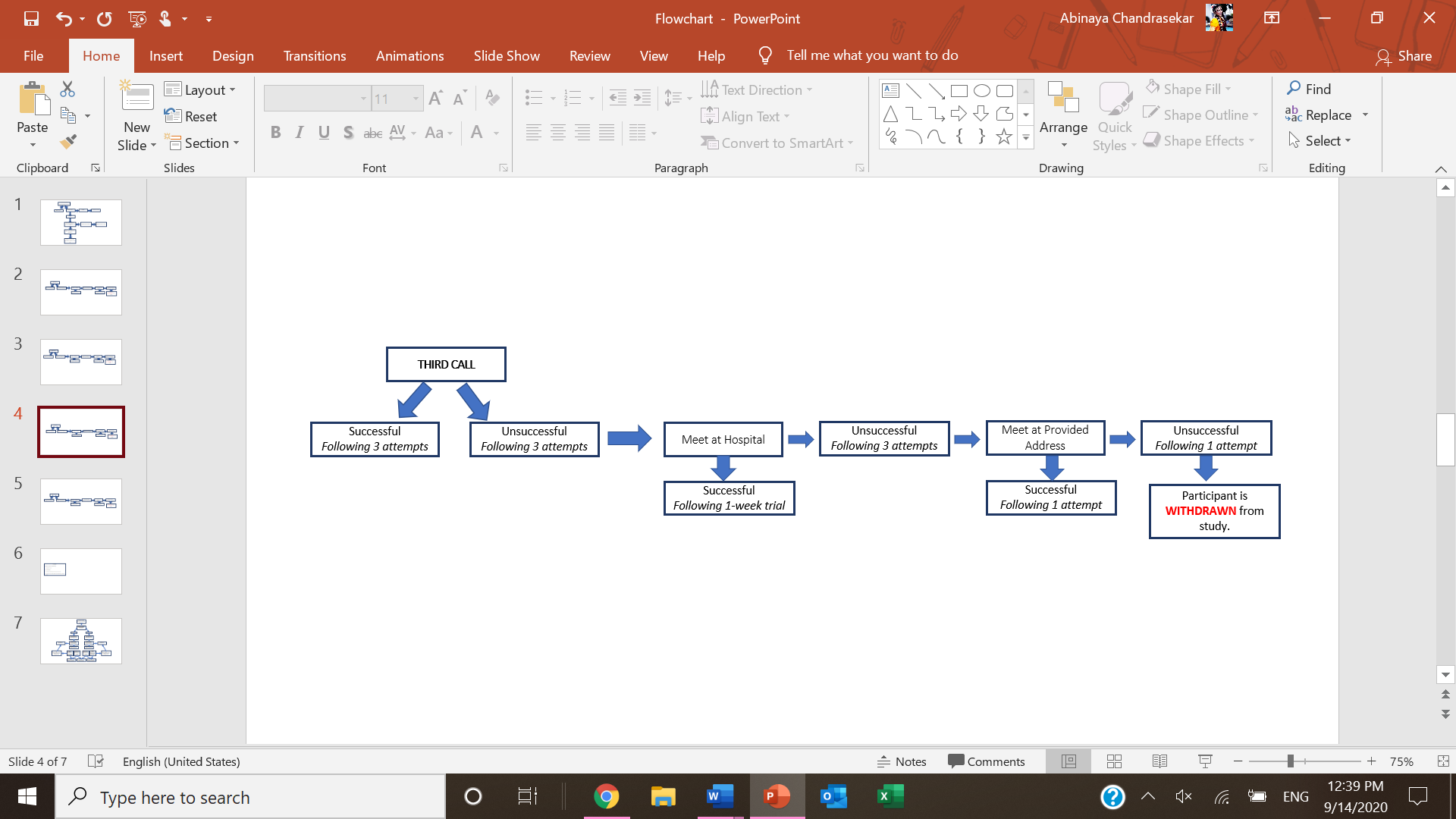

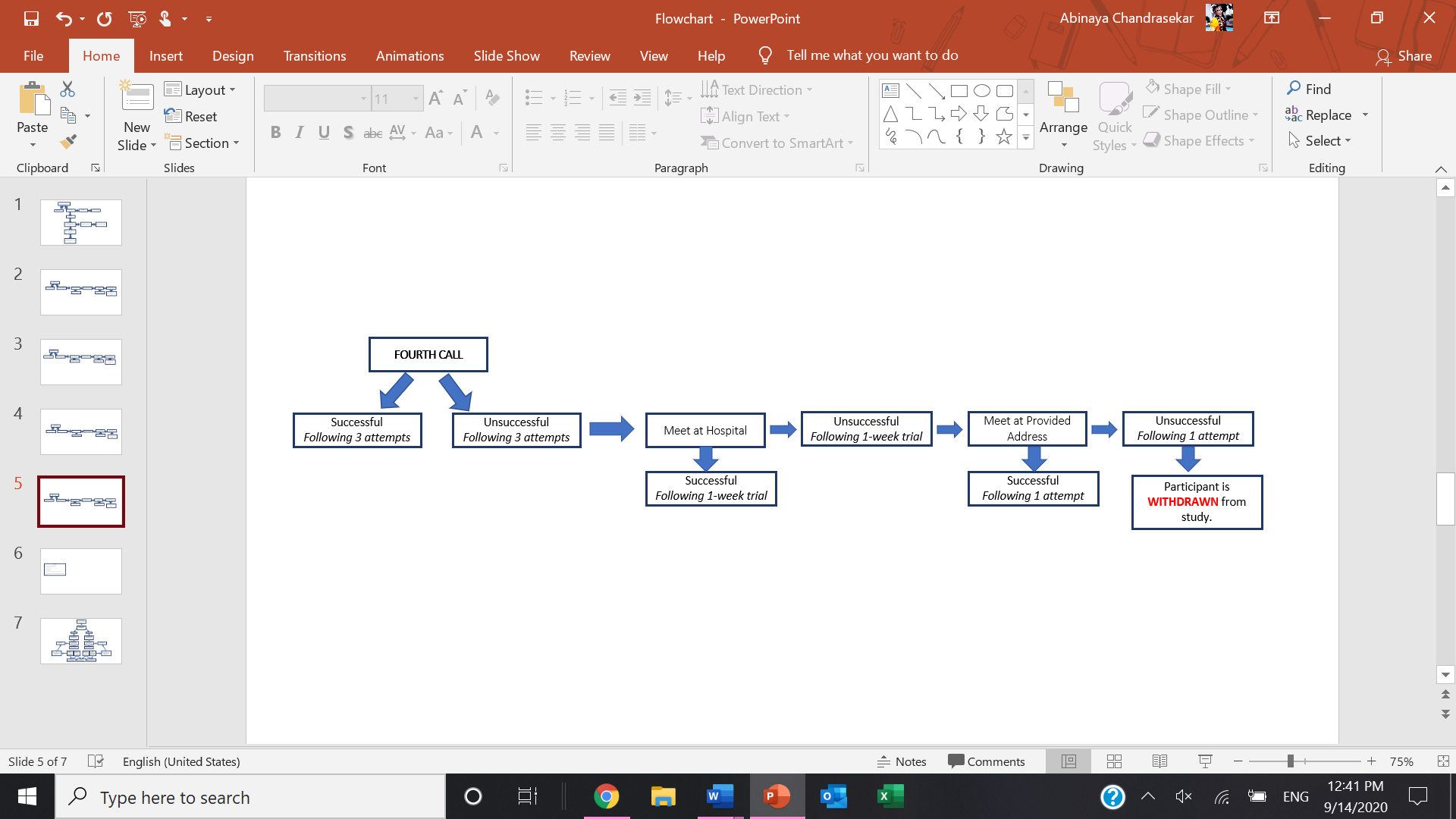

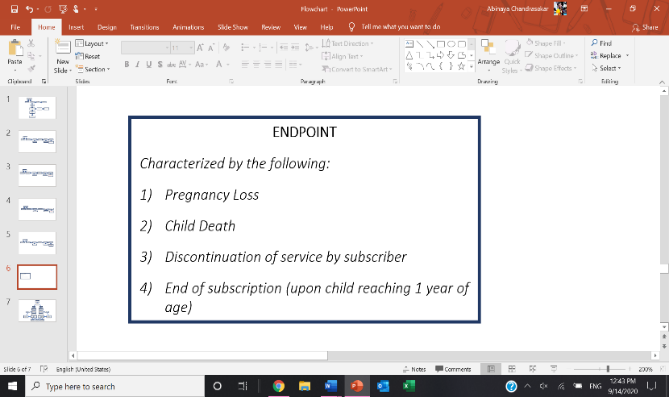


**FLOWCHART OF MMITRA REGISTRATION AND IMPLEMENTATION PROCESS**


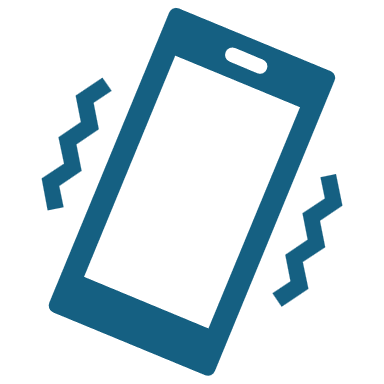

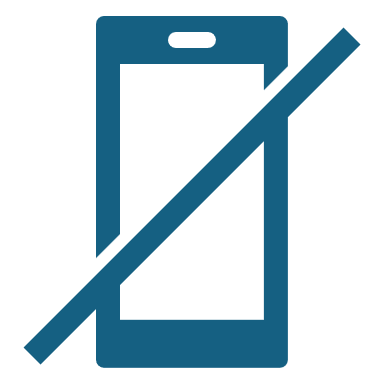


Flowchart of mMitra registration and enrolment process.

1. Murthy N, Chandrasekharan S, Prakash MP, Kaonga NN, Peter J, Ganju A, Mechael PN. The Impact of an mHealth Voice Message Service (mMitra) on Infant Care Knowledge, and Practices Among Low-Income Women in India: Findings from a Pseudo-Randomized Controlled Trial. Maternal and child health journal. 2019;23(12):1658-69.

2. ARMMAN. mMitra 2022 [Available from: <https://www.armman.org/mmitra/>.

3. Mechael P, Kaonga NN, Chandrasekharan S, Prakash MP, Peter J, Ganju A, Murthy N. The Elusive Path Toward Measuring Health Outcomes: Lessons Learned From a Pseudo-Randomized Controlled Trial of a Large-Scale Mobile Health Initiative. JMIR mHealth and uHealth. 2019;7(8):e14668.

4. BabyCenter | Expert info for pregnancy & parenting 2022 [Available from: <https://www.babycenter.com>.

5. Ministry of Health & Family Welfare GoI. A Strategic Approach to Reproductive, Maternal, Newborn, Child and Adolescent Health (RMNCH+A) in India. 2013.
