# Supplementary material for: mHealth interventions for postpartum family planning in LMICs: A realist review
Source: PLOS Glob Public Health. 2024 Jul 18;4(7):e0003432. doi: 10.1371/journal.pgph.0003432 (PMC11257288; doi:10.1371/journal.pgph.0003432)
Supplement: S1 Fig — (DOCX) [file pgph.0003432.s002.docx]

## Appendix B – Dalkin et al. (2015) Heuristic for CMOC representation.


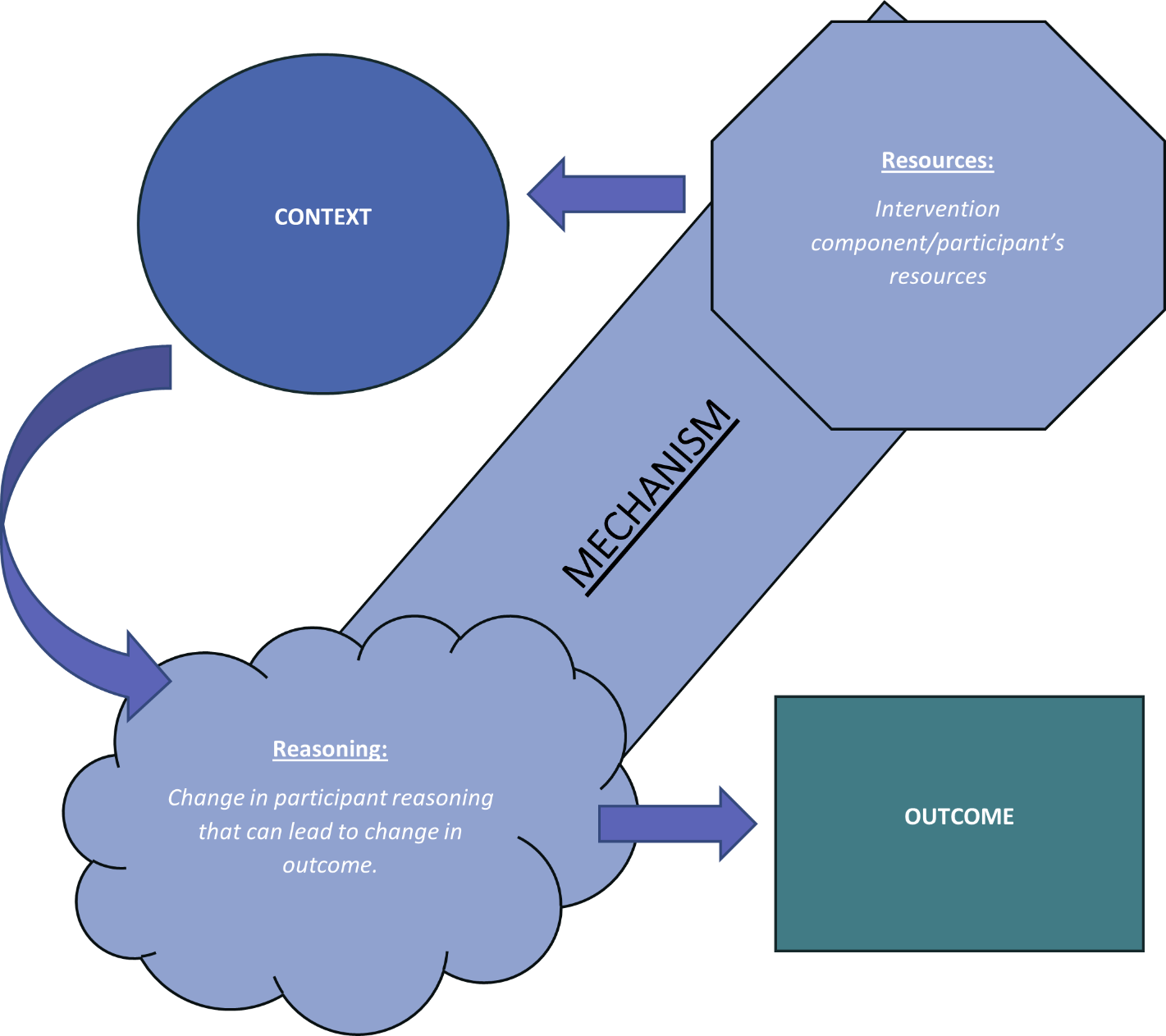


Figure I. CMOC Heuristic proposed by Dalkin et al. (2015)
