# Supplementary material for: mHealth interventions for postpartum family planning in LMICs: A realist review
Source: PLOS Glob Public Health. 2024 Jul 18;4(7):e0003432. doi: 10.1371/journal.pgph.0003432 (PMC11257288; doi:10.1371/journal.pgph.0003432)
Supplement: S1 Table — (DOCX) [file pgph.0003432.s003.docx]

## Appendix C – Realist Review systematic search strategy.

| **Database** | **Search Terms** | **Source/Reference** |
| --- | --- | --- |
| MEDLINE | ((phone adj3 call*) or ((cell* or mobile or smart or google or nexus or iphone) adj3 (phone* or telephone*)) or smartphone* or smart-phone* or (blackberr* not extract) or (black-berr* not extract) or (mobile adj3 health) or mhealth or m-health or e-health* or ehealth* or (electronic* adj1 health) or (mobile adj3 technol*) or ((mobile or smartphone or smart-phone or phone or software) adj3 app*) or MMS or multimedia messaging service or SMS or short messag* service or (text* adj3 messag*) or text-messa* or voice messag* or interactive voice response or IVR or (social adj3 media or network*) or telemedicine or tele-medicine or telehealth or tele-health or telecare or tele-care or mobile app*).mp. | Smith et al. 2015 Allida et al. 2012 Goncalves-Bradley et al. 2012 |
| MEDLINE | exp Telemedicine/ or exp Cell Phone/ or exp Text Messaging/ or exp Internet/ or exp Mobile Applications/ | Smith et al. 2015 Allida et al. 2012 Goncalves-Bradley et al. 2013 |
| MEDLINE | ((condom* or (OC pill) or (depot medroxyprogest* or NET-EN or NET EN or Mesigyna or Cyclofem) or (intrauterine system or intra- uterine system or IUS or intrauterine device* or intra-uterine device* or IUD*) or (vasectomy or sterilisation or sterilization or (tubal ligation)) or ((vaginal ring) or cycletel or cycle-tel or ((abstain or abstinen*) adj2 (sex* or intercourse)) or lactational amenorr*)) or (contracept* or family planning or (birth adj (control or regulat* or spacing)) or planned parenthood or ((population* or fertility) adj3 (regulat* or control))) or (pregnan* adj2 (adolescen* or teen* or schoolchild*)) or (pregnan* adj3 (prevent* or interrupt* or unplanned or unwanted or mistimed))).mp. | Smith et al. 2015 |
| MEDLINE | exp Family Planning Services/ or exp Contraception/ or exp Reproductive Behavior/ or exp Contraception Behavior/ or exp Contraceptive agents/ or exp Contraceptive Devices/ or exp Pregnancy in Adolescence/ or exp Pregnancy, Unplanned/ or exp Pregnancy, Unwanted/ or exp Abortion, Induced/ | Smith et al. 2015 |
| MEDLINE | ((contracept* or (vasectomy or sterilisation or sterilization) or (pregnan* or abortion)) or (NORPLANT or implanon or Femplant)).mp. | Smith et al. 2015 |
| MEDLINE | (postpartum or post-partum or postnatal or post-natal).mp. |  |
| MEDLINE | exp Postpartum Period/ |  |
| MEDLINE | (afghanistan or albania or algeria or "american samoa" or angola or argentina or armenia or armenian or azerbaijan or bangladesh or "republic of belarus" or belarus or byelarus or belorussia or byelorussian or belize or "british honduras" or benin or dahomey or bhutan or bolivia or "bosnia and herzegovina" or bosnia or herzegovina or botswana or bechuanaland or brazil or brasil or bulgaria or "burkina faso" or "burkina fasso" or "upper volta" or burundi or urundi or "cabo verde" or "cape verde" or cambodia or kampuchea or "khmer republic" or cameroon or cameron or cameroun or "central african republic" or "ubangi shari" or chad or china or colombia or comoros or "comoro islands" or "iles comores" or mayotte or "democratic republic of the congo" or "democratic republic congo" or congo or zaire or "costa rica" or "cote d’ivoire" or "cote d’ ivoire" or "cote divoire" or "cote d ivoire" or "ivory coast" or cuba or djibouti or "french somaliland" or dominica or "dominican republic" or ecuador or egypt or "united arab republic" or "el salvador" or "equatorial guinea" or "spanish guinea" or eritrea or eswatini or swaziland or ethiopia or fiji or gabon or "gabonese republic" or gambia or "georgia (republic)" or georgia or georgian or ghana or "gold coast" or grenada or guatemala or guinea or "guinea bissau" or guyana or "british guiana" or haiti or hispaniola or honduras or india or indonesia or timor or iran or iraq or "isle of man" or jamaica or jordan or kazakhstan or kazakh or kenya or kiribati or "democratic people’s republic of korea" or "republic of korea" or north korea or south korea or korea or kosovo or kyrgyzstan or kirghizia or kirgizstan or "kyrgyz republic" or kirghiz or laos or "lao pdr" or "lao people's democratic republic" or lebanon or "lebanese republic" or lesotho or basutoland or liberia or libya or "libyan arab jamahiriya" or madagascar or "malagasy republic" or malawi or nyasaland or malaysia or "malay federation" or "malaya federation" or maldives or "indian ocean islands" or "indian ocean" or mali or micronesia or "federated states of micronesia" or "marshall islands" or nauru or "northern mariana islands" or palau or tuvalu or mauritania or mauritius or mexico or moldova or moldovian or mongolia or montenegro or morocco or ifni or mozambique or "portuguese east africa" or myanmar or burma or namibia or nepal or "netherlands antilles" or nicaragua or niger or nigeria or "republic of north macedonia" or macedonia or north macedonia or pakistan or panama or "papua new guinea" or paraguay or peru or philippines or philipines or phillipines or phillippines or russia or "russian federation" or ussr or "soviet union" or "union of soviet socialist republics" or rwanda or ruanda or samoa or "pacific islands" or polynesia or "samoan islands" or "navigator island" or "navigator islands" or "sao tome and principe" or senegal or serbia or "sierra leone" or melanesia or "solomon island" or "solomon islands" or "norfolk island" or "norfolk islands" or somalia or "south africa" or "south sudan" or "sri lanka" or ceylon or "saint kitts and nevis" or "st. kitts and nevis" or "saint lucia" or "st. lucia" or "saint vincent and the grenadines" or "saint vincent" or "st. vincent" or grenadines or sudan or suriname or surinam or "dutch guiana" or "netherlands guiana" or syria or "syrian arab republic" or tajikistan or tadjikistan or tadzhikistan or tadzhik or tanzania or tanganyika or thailand or siam or "timor leste" or "east timor" or togo or "togolese republic" or tonga or tunisia or turkey or turkmenistan or turkmen or uganda or ukraine or uzbekistan or uzbek or vanuatu or "new hebrides" or venezuela or vietnam or "viet nam" or "middle east" or "west bank" or gaza or palestine or yemen or zambia or zimbabwe or "northern rhodesia" or "global south" or "africa south of the sahara" or "sub saharan africa" or "subsaharan africa" or "africa, central" or "central africa" or "africa, northern" or "north africa" or "northern africa" or magreb or maghrib or sahara or "africa, southern" or "southern africa" or "africa, eastern" or "east africa" or "eastern africa" or "africa, western" or "west africa" or "western africa" or "west indies" or "indian ocean islands" or caribbean or "central america" or "latin america" or "south and central america" or "south america" or "asia, central" or "central asia" or "asia, northern" or "north asia" or "northern asia" or "asia, southeastern" or "southeastern asia" or "south eastern asia" or "southeast asia" or "south east asia" or "asia, western" or "western asia" or "europe, eastern" or "east europe" or "eastern europe" or "developing country" or "developing countries" or "developing nation" or "developing nations" or "developing population" or "developing populations" or "developing world" or "less developed country" or "less developed countries" or "less developed nation" or "less developed nations" or "less developed population" or "less developed populations" or "less developed world" or "lesser developed country" or "lesser developed countries" or "lesser developed nation" or "lesser developed nations" or "lesser developed population" or "lesser developed populations" or "lesser developed world" or "under developed country" or "under developed countries" or "under developed nation" or "under developed nations" or "under developed population" or "under developed populations" or "under developed world" or "underdeveloped country" or "underdeveloped countries" or "underdeveloped nation" or "underdeveloped nations" or "underdeveloped population" or "underdeveloped populations" or "underdeveloped world" or "middle income country" or "middle income countries" or "middle income nation" or "middle income nations" or "middle income population" or "middle income populations" or "low income country" or "low income countries" or "low income nation" or "low income nations" or "low income population" or "low income populations" or "lower income country" or "lower income countries" or "lower income nation" or "lower income nations" or "lower income population" or "lower income populations" or "underserved country" or "underserved countries" or "underserved nation" or "underserved nations" or "underserved population" or "underserved populations" or "underserved world" or "under served country" or "under served countries" or "under served nation" or "under served nations" or "under served population" or "under served populations" or "under served world" or "deprived country" or "deprived countries" or "deprived nation" or "deprived nations" or "deprived population" or "deprived populations" or "deprived world" or "poor country" or "poor countries" or "poor nation" or "poor nations" or "poor population" or "poor populations" or "poor world" or "poorer country" or "poorer countries" or "poorer nation" or "poorer nations" or "poorer population" or "poorer populations" or "poorer world" or "developing economy" or "developing economies" or "less developed economy" or "less developed economies" or "lesser developed economy" or "lesser developed economies" or "under developed economy" or "under developed economies" or "underdeveloped economy" or "underdeveloped economies" or "middle income economy" or "middle income economies" or "low income economy" or "low income economies" or "lower income economy" or "lower income economies" or "low gdp" or "low gnp" or "low gross domestic" or "low gross national" or "lower gdp" or "lower gnp" or "lower gross domestic" or "lower gross national" or lmic or lmics or "third world" or "lami country" or "lami countries" or "transitional country" or "transitional countries" or "emerging economies" or "emerging nation" or "emerging nations").mp. |  |
| EMBASE | ((phone adj3 call*) OR ((cell* or mobile or smart or google or nexus or iphone) adj3 (phone* or telephone*)) OR smartphone* OR smart-phone* OR (blackberr* not extract) OR (black-berr* not extract) OR mhealth OR m-health OR e-health* OR ehealth* OR (electronic adj health) OR (mobile adj3 technol*) OR ((mobileor smartphone or smart-phone or phone or software) adj3 app*) OR MMS OR multimedia messaging service OR SMS OR short messag* service OR (text* adj messag*) OR text-messa* OR voice messag* OR interactive voice response OR IVR).mp. | Smith et al. 2015 |
| EMBASE | Telemedicine/ OR cellular phone/ OR text messaging/ | Smith et al. 2015 |
| EMBASE | ((contracept* or (family planning) or (Birth control)) OR condom OR (OC pill) OR (depot medroxyprogest* or NET-EN or NET EN or Mesigyna or Cyclofem) OR (intrauterine system or intra-uterine system or IUS or intrauterine device or intra-uterinedevice or IUD) OR (vasectomy or sterilisation or sterilization or (tubal ligation)) OR ((vaginal ring) or cycletel or cycle-telor abstain or abstinen* or lactational amenorr*) OR (pregnan* or abortion) OR (NORPLANT or implanon or Femplant)).mp. | Smith et al. 2015 |
| EMBASE | exp vagina contraception/ or exp emergency contraception/ or exp barrier contraception/ or exp contraception/ or exp long-acting reversible contraception/ or exp hormonal contraception/ or exp oral contraception/ or exp contraceptive device/ or exp unplanned pregnnacy/ or exp unwanted pregnancy/ or exp induced abortion/ or exp reproductive health/ | Smith et al. 2015 |
| EMBASE | ((contracept* or family planning or (birth adj (control or regulat* or spacing)) or planned parenthood or ((population or fertility) adj(regulat* or control)))or (pregnan* adj3 (prevent* or interrupt* or unplanned or unwanted or mistimed)) or (abort* or miscarr* or (pregnan* adj2 terminat*)) or (reproductive adj2 (health or care or service*))).mp. | Palmer et al. 2020 |
| EMBASE | exp family planning/ or reproductive behavior/ or contraceptive behavior/ or exp contraceptive agent/ | Palmer et al. 2020 |
| EMBASE | (postnatal or post-natal or post$natal or postpartum or post-partum or post$partum or ((postnatal or post-natal or perinatal or peri-natal or postpartum or post-partum) adj2 (care or service*))).mp. |  |
| EMBASE | exp puerperium/ or postnatal care/ |  |
| EMBASE | (afghanistan or albania or algeria or "american samoa" or angola or argentina or armenia or armenian or azerbaijan or bangladesh or "republic of belarus" or belarus or byelarus or belorussia or byelorussian or belize or "british honduras" or benin or dahomey or bhutan or bolivia or "bosnia and herzegovina" or bosnia or herzegovina or botswana or bechuanaland or brazil or brasil or bulgaria or "burkina faso" or "burkina fasso" or "upper volta" or burundi or urundi or "cabo verde" or "cape verde" or cambodia or kampuchea or "khmer republic" or cameroon or cameron or cameroun or "central african republic" or "ubangi shari" or chad or china or colombia or comoros or "comoro islands" or "iles comores" or mayotte or "democratic republic of the congo" or "democratic republic congo" or congo or zaire or "costa rica" or "cote d’ivoire" or "cote d’ ivoire" or "cote divoire" or "cote d ivoire" or "ivory coast" or cuba or djibouti or "french somaliland" or dominica or "dominican republic" or ecuador or egypt or "united arab republic" or "el salvador" or "equatorial guinea" or "spanish guinea" or eritrea or eswatini or swaziland or ethiopia or fiji or gabon or "gabonese republic" or gambia or "georgia (republic)" or georgia or georgian or ghana or "gold coast" or grenada or guatemala or guinea or "guinea bissau" or guyana or "british guiana" or haiti or hispaniola or honduras or india or indonesia or timor or iran or iraq or "isle of man" or jamaica or jordan or kazakhstan or kazakh or kenya or kiribati or "democratic people’s republic of korea" or "republic of korea" or north korea or south korea or korea or kosovo or kyrgyzstan or kirghizia or kirgizstan or "kyrgyz republic" or kirghiz or laos or "lao pdr" or "lao people's democratic republic" or lebanon or "lebanese republic" or lesotho or basutoland or liberia or libya or "libyan arab jamahiriya" or madagascar or "malagasy republic" or malawi or nyasaland or malaysia or "malay federation" or "malaya federation" or maldives or "indian ocean islands" or "indian ocean" or mali or micronesia or "federated states of micronesia" or "marshall islands" or nauru or "northern mariana islands" or palau or tuvalu or mauritania or mauritius or mexico or moldova or moldovian or mongolia or montenegro or morocco or ifni or mozambique or "portuguese east africa" or myanmar or burma or namibia or nepal or "netherlands antilles" or nicaragua or niger or nigeria or "republic of north macedonia" or macedonia or north macedonia or pakistan or panama or "papua new guinea" or paraguay or peru or philippines or philipines or phillipines or phillippines or russia or "russian federation" or ussr or "soviet union" or "union of soviet socialist republics" or rwanda or ruanda or samoa or "pacific islands" or polynesia or "samoan islands" or "navigator island" or "navigator islands" or "sao tome and principe" or senegal or serbia or "sierra leone" or melanesia or "solomon island" or "solomon islands" or "norfolk island" or "norfolk islands" or somalia or "south africa" or "south sudan" or "sri lanka" or ceylon or "saint kitts and nevis" or "st. kitts and nevis" or "saint lucia" or "st. lucia" or "saint vincent and the grenadines" or "saint vincent" or "st. vincent" or grenadines or sudan or suriname or surinam or "dutch guiana" or "netherlands guiana" or syria or "syrian arab republic" or tajikistan or tadjikistan or tadzhikistan or tadzhik or tanzania or tanganyika or thailand or siam or "timor leste" or "east timor" or togo or "togolese republic" or tonga or tunisia or turkey or turkmenistan or turkmen or uganda or ukraine or uzbekistan or uzbek or vanuatu or "new hebrides" or venezuela or vietnam or "viet nam" or "middle east" or "west bank" or gaza or palestine or yemen or zambia or zimbabwe or "northern rhodesia" or "global south" or "africa south of the sahara" or "sub saharan africa" or "subsaharan africa" or "africa, central" or "central africa" or "africa, northern" or "north africa" or "northern africa" or magreb or maghrib or sahara or "africa, southern" or "southern africa" or "africa, eastern" or "east africa" or "eastern africa" or "africa, western" or "west africa" or "western africa" or "west indies" or "indian ocean islands" or caribbean or "central america" or "latin america" or "south and central america" or "south america" or "asia, central" or "central asia" or "asia, northern" or "north asia" or "northern asia" or "asia, southeastern" or "southeastern asia" or "south eastern asia" or "southeast asia" or "south east asia" or "asia, western" or "western asia" or "europe, eastern" or "east europe" or "eastern europe" or "developing country" or "developing countries" or "developing nation" or "developing nations" or "developing population" or "developing populations" or "developing world" or "less developed country" or "less developed countries" or "less developed nation" or "less developed nations" or "less developed population" or "less developed populations" or "less developed world" or "lesser developed country" or "lesser developed countries" or "lesser developed nation" or "lesser developed nations" or "lesser developed population" or "lesser developed populations" or "lesser developed world" or "under developed country" or "under developed countries" or "under developed nation" or "under developed nations" or "under developed population" or "under developed populations" or "under developed world" or "underdeveloped country" or "underdeveloped countries" or "underdeveloped nation" or "underdeveloped nations" or "underdeveloped population" or "underdeveloped populations" or "underdeveloped world" or "middle income country" or "middle income countries" or "middle income nation" or "middle income nations" or "middle income population" or "middle income populations" or "low income country" or "low income countries" or "low income nation" or "low income nations" or "low income population" or "low income populations" or "lower income country" or "lower income countries" or "lower income nation" or "lower income nations" or "lower income population" or "lower income populations" or "underserved country" or "underserved countries" or "underserved nation" or "underserved nations" or "underserved population" or "underserved populations" or "underserved world" or "under served country" or "under served countries" or "under served nation" or "under served nations" or "under served population" or "under served populations" or "under served world" or "deprived country" or "deprived countries" or "deprived nation" or "deprived nations" or "deprived population" or "deprived populations" or "deprived world" or "poor country" or "poor countries" or "poor nation" or "poor nations" or "poor population" or "poor populations" or "poor world" or "poorer country" or "poorer countries" or "poorer nation" or "poorer nations" or "poorer population" or "poorer populations" or "poorer world" or "developing economy" or "developing economies" or "less developed economy" or "less developed economies" or "lesser developed economy" or "lesser developed economies" or "under developed economy" or "under developed economies" or "underdeveloped economy" or "underdeveloped economies" or "middle income economy" or "middle income economies" or "low income economy" or "low income economies" or "lower income economy" or "lower income economies" or "low gdp" or "low gnp" or "low gross domestic" or "low gross national" or "lower gdp" or "lower gnp" or "lower gross domestic" or "lower gross national" or lmic or lmics or "third world" or "lami country" or "lami countries" or "transitional country" or "transitional countries" or "emerging economies" or "emerging nation" or "emerging nations").mp. | Cochrane LMIC Filter - Central |
| Global Health | ((phone adj3 call*) OR ((cell* or mobile or smart or google or nexus or iphone) adj3 (phone* or telephone*)) OR smartphone* OR smart-phone* OR (blackberr* not extract) OR (black-berr* not extract) OR (mobile adj3 health) OR mhealth OR m-health OR e-health* OR ehealth* OR (electronic* adj1 health) OR (mobile adj3 technol*) OR ((mobile or smartphone or smart-phone or phone or software) adj3 app*) OR MMS OR multimedia messaging service OR SMS OR short messag* service OR (text* adj3 messag*) OR text-messa* OR voice messag* OR interactive voice response OR IVR).mp. | Smith et al. 2015 |
| Global Health | telemedicine/ OR text messaging/ OR mobile telephones/ | Smith et al. 2015 |
| Global Health | ((contracept* or (family planning) or (Birth control)) OR condom OR (OC pill) OR (depot medroxyprogest* or NET-EN or NET EN or Mesigyna or Cyclofem) OR (intrauterine system or intra-uterine system or IUS or intrauterine device or intra-uterinedevice or IUD) OR (vasectomy or sterilisation or sterilization or (tubal ligation)) OR ((vaginal ring) or cycletel or cycle-telor abstain or abstinen* or lactational amenorr*) OR (pregnan* or abortion) OR (NORPLANT or implanon or Femplant)).mp. | Smith et al. 2015 |
| Global Health | exp contraception/ OR exp contraceptives/ or exp sexual behaviour/ or exp intrauterine devices/ or exp family planning/ or exp induced abortion/ | Smith et al. 2015 |
| Global Health | ((contracept* or family planning or (birth adj (control or regulat* or spacing)) or planned parenthood or ((population or fertility) adj(regulat* or control)))or (pregnan* adj3 (prevent* or interrupt* or unplanned or unwanted or mistimed)) or (abort* or miscarr* or (pregnan* adj2 terminat*)) or (reproductive adj2 (health or care or service*))).mp. | Smith et al. 2015 |
| Global Health | (postpartum or post-partum or postnatal or post-natal).mp. |  |
| Global Health | exp postpartum period/ or exp postpartum interval/ or exp puerperium/ |  |
| Global Health | (afghanistan or albania or algeria or "american samoa" or angola or argentina or armenia or armenian or azerbaijan or bangladesh or "republic of belarus" or belarus or byelarus or belorussia or byelorussian or belize or "british honduras" or benin or dahomey or bhutan or bolivia or "bosnia and herzegovina" or bosnia or herzegovina or botswana or bechuanaland or brazil or brasil or bulgaria or "burkina faso" or "burkina fasso" or "upper volta" or burundi or urundi or "cabo verde" or "cape verde" or cambodia or kampuchea or "khmer republic" or cameroon or cameron or cameroun or "central african republic" or "ubangi shari" or chad or china or colombia or comoros or "comoro islands" or "iles comores" or mayotte or "democratic republic of the congo" or "democratic republic congo" or congo or zaire or "costa rica" or "cote d’ivoire" or "cote d’ ivoire" or "cote divoire" or "cote d ivoire" or "ivory coast" or cuba or djibouti or "french somaliland" or dominica or "dominican republic" or ecuador or egypt or "united arab republic" or "el salvador" or "equatorial guinea" or "spanish guinea" or eritrea or eswatini or swaziland or ethiopia or fiji or gabon or "gabonese republic" or gambia or "georgia (republic)" or georgia or georgian or ghana or "gold coast" or grenada or guatemala or guinea or "guinea bissau" or guyana or "british guiana" or haiti or hispaniola or honduras or india or indonesia or timor or iran or iraq or "isle of man" or jamaica or jordan or kazakhstan or kazakh or kenya or kiribati or "democratic people’s republic of korea" or "republic of korea" or north korea or south korea or korea or kosovo or kyrgyzstan or kirghizia or kirgizstan or "kyrgyz republic" or kirghiz or laos or "lao pdr" or "lao people's democratic republic" or lebanon or "lebanese republic" or lesotho or basutoland or liberia or libya or "libyan arab jamahiriya" or madagascar or "malagasy republic" or malawi or nyasaland or malaysia or "malay federation" or "malaya federation" or maldives or "indian ocean islands" or "indian ocean" or mali or micronesia or "federated states of micronesia" or "marshall islands" or nauru or "northern mariana islands" or palau or tuvalu or mauritania or mauritius or mexico or moldova or moldovian or mongolia or montenegro or morocco or ifni or mozambique or "portuguese east africa" or myanmar or burma or namibia or nepal or "netherlands antilles" or nicaragua or niger or nigeria or "republic of north macedonia" or macedonia or north macedonia or pakistan or panama or "papua new guinea" or paraguay or peru or philippines or philipines or phillipines or phillippines or russia or "russian federation" or ussr or "soviet union" or "union of soviet socialist republics" or rwanda or ruanda or samoa or "pacific islands" or polynesia or "samoan islands" or "navigator island" or "navigator islands" or "sao tome and principe" or senegal or serbia or "sierra leone" or melanesia or "solomon island" or "solomon islands" or "norfolk island" or "norfolk islands" or somalia or "south africa" or "south sudan" or "sri lanka" or ceylon or "saint kitts and nevis" or "st. kitts and nevis" or "saint lucia" or "st. lucia" or "saint vincent and the grenadines" or "saint vincent" or "st. vincent" or grenadines or sudan or suriname or surinam or "dutch guiana" or "netherlands guiana" or syria or "syrian arab republic" or tajikistan or tadjikistan or tadzhikistan or tadzhik or tanzania or tanganyika or thailand or siam or "timor leste" or "east timor" or togo or "togolese republic" or tonga or tunisia or turkey or turkmenistan or turkmen or uganda or ukraine or uzbekistan or uzbek or vanuatu or "new hebrides" or venezuela or vietnam or "viet nam" or "middle east" or "west bank" or gaza or palestine or yemen or zambia or zimbabwe or "northern rhodesia" or "global south" or "africa south of the sahara" or "sub saharan africa" or "subsaharan africa" or "africa, central" or "central africa" or "africa, northern" or "north africa" or "northern africa" or magreb or maghrib or sahara or "africa, southern" or "southern africa" or "africa, eastern" or "east africa" or "eastern africa" or "africa, western" or "west africa" or "western africa" or "west indies" or "indian ocean islands" or caribbean or "central america" or "latin america" or "south and central america" or "south america" or "asia, central" or "central asia" or "asia, northern" or "north asia" or "northern asia" or "asia, southeastern" or "southeastern asia" or "south eastern asia" or "southeast asia" or "south east asia" or "asia, western" or "western asia" or "europe, eastern" or "east europe" or "eastern europe" or "developing country" or "developing countries" or "developing nation" or "developing nations" or "developing population" or "developing populations" or "developing world" or "less developed country" or "less developed countries" or "less developed nation" or "less developed nations" or "less developed population" or "less developed populations" or "less developed world" or "lesser developed country" or "lesser developed countries" or "lesser developed nation" or "lesser developed nations" or "lesser developed population" or "lesser developed populations" or "lesser developed world" or "under developed country" or "under developed countries" or "under developed nation" or "under developed nations" or "under developed population" or "under developed populations" or "under developed world" or "underdeveloped country" or "underdeveloped countries" or "underdeveloped nation" or "underdeveloped nations" or "underdeveloped population" or "underdeveloped populations" or "underdeveloped world" or "middle income country" or "middle income countries" or "middle income nation" or "middle income nations" or "middle income population" or "middle income populations" or "low income country" or "low income countries" or "low income nation" or "low income nations" or "low income population" or "low income populations" or "lower income country" or "lower income countries" or "lower income nation" or "lower income nations" or "lower income population" or "lower income populations" or "underserved country" or "underserved countries" or "underserved nation" or "underserved nations" or "underserved population" or "underserved populations" or "underserved world" or "under served country" or "under served countries" or "under served nation" or "under served nations" or "under served population" or "under served populations" or "under served world" or "deprived country" or "deprived countries" or "deprived nation" or "deprived nations" or "deprived population" or "deprived populations" or "deprived world" or "poor country" or "poor countries" or "poor nation" or "poor nations" or "poor population" or "poor populations" or "poor world" or "poorer country" or "poorer countries" or "poorer nation" or "poorer nations" or "poorer population" or "poorer populations" or "poorer world" or "developing economy" or "developing economies" or "less developed economy" or "less developed economies" or "lesser developed economy" or "lesser developed economies" or "under developed economy" or "under developed economies" or "underdeveloped economy" or "underdeveloped economies" or "middle income economy" or "middle income economies" or "low income economy" or "low income economies" or "lower income economy" or "lower income economies" or "low gdp" or "low gnp" or "low gross domestic" or "low gross national" or "lower gdp" or "lower gnp" or "lower gross domestic" or "lower gross national" or lmic or lmics or "third world" or "lami country" or "lami countries" or "transitional country" or "transitional countries" or "emerging economies" or "emerging nation" or "emerging nations").mp. | Cochrane LMIC filter - Central |
| Web of Science | TI=((phone NEAR/3 call*) or ((cell* or mobile or smart or google or nexus or iphone) NEAR/3 (phone* or telephone*)) or smartphone* or smart-phone* or (blackberr* not extract) or (black-berr* not extract) or (mobile NEAR/3 health) or mhealth or m-health or e-health* or ehealth* or (electronic* NEAR/1 health) or (mobile NEAR/3 technol*) or ((mobile or smartphone or smart-phone or phone or software) NEAR/3 app*) or MMS or multimedia messaging service or SMS or short messag* service or (text* NEAR/3 messag*)) OR AB=((phone NEAR/3 call*) or ((cell* or mobile or smart or google or nexus or iphone) NEAR/3 (phone* or telephone*)) or smartphone* or smart-phone* or (blackberr* not extract) or (black-berr* not extract) or (mobile NEAR/3 health) or mhealth or m-health or e-health* or ehealth* or (electronic* NEAR/1 health) or (mobile NEAR/3 technol*) or ((mobile or smartphone or smart-phone or phone or software) NEAR/3 app*) or MMS or multimedia messaging service or SMS or short messag* service or (text* NEAR/3 messag*)) | Smith et al. 2015  Allida et al. 2012  Goncalves-Bradley et al. 2012 |
| Web of Science | TI=((text-messa* or voice messag* or interactive voice response or IVR or (social NEAR/3 media or network*) or telemedicine or tele-medicine or telehealth or tele-health or telecare or tele-care or mobile app*)) OR AB=((text-messa* or voice messag* or interactive voice response or IVR or (social NEAR/3 media or network*) or telemedicine or tele-medicine or telehealth or tele-health or telecare or tele-care or mobile app*)) | Smith et al. 2015  Allida et al. 2012  Goncalves-Bradley et al. 2012 |
| Web of Science | TS=(telemedicine or cell phone or text messaging or internet or mobile applications) | Smith et al. 2015  Allida et al. 2012  Goncalves-Bradley et al. 2012 |
| Web of Science | TI=((condom* or (OC pill) or (depot medroxyprogest* or NET-EN or NET EN or Mesigyna or Cyclofem) or (intrauterine system or intra- uterine system or IUS or intrauterine device* or intra-uterine device* or IUD*) or (vasectomy or sterilisation or sterilization or (tubal ligation)) or ((vaginal ring) or cycletel or cycle-tel or ((abstain or abstinen*) NEAR/2 (sex* or intercourse)) or lactational amenorr*))) OR AB=((condom* or (OC pill) or (depot medroxyprogest* or NET-EN or NET EN or Mesigyna or Cyclofem) or (intrauterine system or intra- uterine system or IUS or intrauterine device* or intra-uterine device* or IUD*) or (vasectomy or sterilisation or sterilization or (tubal ligation)) or ((vaginal ring) or cycletel or cycle-tel or ((abstain or abstinen*) NEAR/2 (sex* or intercourse)) or lactational amenorr*))) | Smith et al. 2015 |
| Web of Science | TI=(((contracept* or family planning or (birth NEAR (control or regulat* or spacing)) or planned parenthood or ((population* or fertility) NEAR/3 (regulat* or control))) or (pregnan* NEAR/2 (adolescen* or teen* or schoolchild*)) or (pregnan* NEAR/3 (prevent* or interrupt* or unplanned or unwanted or mistimed)))) OR AB=(((contracept* or family planning or (birth NEAR (control or regulat* or spacing)) or planned parenthood or ((population* or fertility) NEAR/3 (regulat* or control))) or (pregnan* NEAR/2 (adolescen* or teen* or schoolchild*)) or (pregnan* NEAR/3 (prevent* or interrupt* or unplanned or unwanted or mistimed)))) | Smith et al. 2015 |
| Web of Science | TI=((contracept* or (vasectomy or sterilisation or sterilization) or (pregnan* or abortion)) or (NORPLANT or implanon or Femplant)) OR AB=((contracept* or (vasectomy or sterilisation or sterilization) or (pregnan* or abortion)) or (NORPLANT or implanon or Femplant)) | Smith et al. 2015 |
| Web of Science | TI=(postpartum or post-partum or postnatal or post-natal or puerperium) OR AB=(postpartum or post-partum or postnatal or post-natal or puerperium) |  |
| Web of Science | TI=(afghanistan or albania or algeria or "american samoa" or angola or argentina or armenia or armenian or azerbaijan or bangladesh or "republic of belarus" or belarus or byelarus or belorussia or byelorussian or belize or "british honduras" or benin or dahomey or bhutan or bolivia or "bosnia and herzegovina" or bosnia or herzegovina or botswana or bechuanaland or brazil or brasil or bulgaria or "burkina faso" or "burkina fasso" or "upper volta" or burundi or urundi or "cabo verde" or "cape verde" or cambodia or kampuchea or "khmer republic" or cameroon or cameron or cameroun or "central african republic" or "ubangi shari" or chad or china or colombia or comoros or "comoro islands" or "iles comores" or mayotte or "democratic republic of the congo" or "democratic republic congo" or congo or zaire or "costa rica" or "cote d’ivoire" or "cote d’ ivoire" or "cote divoire" or "cote d ivoire" or "ivory coast" or cuba or djibouti or "french somaliland" or dominica or "dominican republic" or ecuador or egypt or "united arab republic" or "el salvador" or "equatorial guinea" or "spanish guinea" or eritrea or eswatini or swaziland or ethiopia or fiji or gabon or "gabonese republic" or gambia or "georgia (republic)" or georgia or georgian or ghana or "gold coast" or grenada or guatemala or guinea or "guinea bissau" or guyana or "british guiana" or haiti or hispaniola or honduras or india or indonesia or timor or iran or iraq or "isle of man" or jamaica or jordan or kazakhstan or kazakh or kenya or kiribati or "democratic people’s republic of korea" or "republic of korea" or north korea or south korea or korea or kosovo or kyrgyzstan or kirghizia or kirgizstan or "kyrgyz republic" or kirghiz or laos or "lao pdr" or "lao people's democratic republic" or lebanon or "lebanese republic" or lesotho or basutoland or liberia or libya or "libyan arab jamahiriya" or madagascar or "malagasy republic" or malawi or nyasaland or malaysia or "malay federation" or "malaya federation" or maldives or "indian ocean islands" or "indian ocean" or mali or micronesia or "federated states of micronesia" or "marshall islands" or nauru or "northern mariana islands" or palau or tuvalu or mauritania or mauritius or mexico or moldova or moldovian or mongolia or montenegro or morocco or ifni or mozambique or "portuguese east africa" or myanmar or burma or namibia or nepal or "netherlands antilles" or nicaragua or niger or nigeria or "republic of north macedonia" or macedonia or north macedonia or pakistan or panama or "papua new guinea" or paraguay or peru or philippines or philipines or phillipines or phillippines or russia or "russian federation" or ussr or "soviet union" or "union of soviet socialist republics" or rwanda or ruanda or samoa or "pacific islands" or polynesia or "samoan islands" or "navigator island" or "navigator islands" or "sao tome and principe" or senegal or serbia or "sierra leone" or melanesia or "solomon island" or "solomon islands" or "norfolk island" or "norfolk islands" or somalia or "south africa" or "south sudan" or "sri lanka" or ceylon or "saint kitts and nevis" or "st. kitts and nevis" or "saint lucia" or "st. lucia" or "saint vincent and the grenadines" or "saint vincent" or "st. vincent" or grenadines or sudan or suriname or surinam or "dutch guiana" or "netherlands guiana" or syria or "syrian arab republic" or tajikistan or tadjikistan or tadzhikistan or tadzhik or tanzania or tanganyika or thailand or siam or "timor leste" or "east timor" or togo or "togolese republic" or tonga or tunisia or turkey or turkmenistan or turkmen or uganda or ukraine or uzbekistan or uzbek or vanuatu or "new hebrides" or venezuela or vietnam or "viet nam" or "middle east" or "west bank" or gaza or palestine or yemen or zambia or zimbabwe or "northern rhodesia" or "global south" or "africa south of the sahara" or "sub saharan africa" or "subsaharan africa" or "africa, central" or "central africa" or "africa, northern" or "north africa" or "northern africa" or magreb or maghrib or sahara or "africa, southern" or "southern africa" or "africa, eastern" or "east africa" or "eastern africa" or "africa, western" or "west africa" or "western africa" or "west indies" or "indian ocean islands" or caribbean or "central america" or "latin america" or "south and central america" or "south america" or "asia, central" or "central asia" or "asia, northern" or "north asia" or "northern asia" or "asia, southeastern" or "southeastern asia" or "south eastern asia" or "southeast asia" or "south east asia" or "asia, western" or "western asia" or "europe, eastern" or "east europe" or "eastern europe" or "developing country" or "developing countries" or "developing nation" or "developing nations" or "developing population" or "developing populations" or "developing world" or "less developed country" or "less developed countries" or "less developed nation" or "less developed nations" or "less developed population" or "less developed populations" or "less developed world" or "lesser developed country" or "lesser developed countries" or "lesser developed nation" or "lesser developed nations" or "lesser developed population" or "lesser developed populations" or "lesser developed world" or "under developed country" or "under developed countries" or "under developed nation" or "under developed nations" or "under developed population" or "under developed populations" or "under developed world" or "underdeveloped country" or "underdeveloped countries" or "underdeveloped nation" or "underdeveloped nations" or "underdeveloped population" or "underdeveloped populations" or "underdeveloped world" or "middle income country" or "middle income countries" or "middle income nation" or "middle income nations" or "middle income population" or "middle income populations" or "low income country" or "low income countries" or "low income nation" or "low income nations" or "low income population" or "low income populations" or "lower income country" or "lower income countries" or "lower income nation" or "lower income nations" or "lower income population" or "lower income populations" or "underserved country" or "underserved countries" or "underserved nation" or "underserved nations" or "underserved population" or "underserved populations" or "underserved world" or "under served country" or "under served countries" or "under served nation" or "under served nations" or "under served population" or "under served populations" or "under served world" or "deprived country" or "deprived countries" or "deprived nation" or "deprived nations" or "deprived population" or "deprived populations" or "deprived world" or "poor country" or "poor countries" or "poor nation" or "poor nations" or "poor population" or "poor populations" or "poor world" or "poorer country" or "poorer countries" or "poorer nation" or "poorer nations" or "poorer population" or "poorer populations" or "poorer world" or "developing economy" or "developing economies" or "less developed economy" or "less developed economies" or "lesser developed economy" or "lesser developed economies" or "under developed economy" or "under developed economies" or "underdeveloped economy" or "underdeveloped economies" or "middle income economy" or "middle income economies" or "low income economy" or "low income economies" or "lower income economy" or "lower income economies" or "low gdp" or "low gnp" or "low gross domestic" or "low gross national" or "lower gdp" or "lower gnp" or "lower gross domestic" or "lower gross national" or lmic or lmics or "third world" or "lami country" or "lami countries" or "transitional country" or "transitional countries" or "emerging economies" or "emerging nation" or "emerging nations") | Cochrane LMIC Filter - Central |
| Web of Science | AB=(afghanistan or albania or algeria or "american samoa" or angola or argentina or armenia or armenian or azerbaijan or bangladesh or "republic of belarus" or belarus or byelarus or belorussia or byelorussian or belize or "british honduras" or benin or dahomey or bhutan or bolivia or "bosnia and herzegovina" or bosnia or herzegovina or botswana or bechuanaland or brazil or brasil or bulgaria or "burkina faso" or "burkina fasso" or "upper volta" or burundi or urundi or "cabo verde" or "cape verde" or cambodia or kampuchea or "khmer republic" or cameroon or cameron or cameroun or "central african republic" or "ubangi shari" or chad or china or colombia or comoros or "comoro islands" or "iles comores" or mayotte or "democratic republic of the congo" or "democratic republic congo" or congo or zaire or "costa rica" or "cote d’ivoire" or "cote d’ ivoire" or "cote divoire" or "cote d ivoire" or "ivory coast" or cuba or djibouti or "french somaliland" or dominica or "dominican republic" or ecuador or egypt or "united arab republic" or "el salvador" or "equatorial guinea" or "spanish guinea" or eritrea or eswatini or swaziland or ethiopia or fiji or gabon or "gabonese republic" or gambia or "georgia (republic)" or georgia or georgian or ghana or "gold coast" or grenada or guatemala or guinea or "guinea bissau" or guyana or "british guiana" or haiti or hispaniola or honduras or india or indonesia or timor or iran or iraq or "isle of man" or jamaica or jordan or kazakhstan or kazakh or kenya or kiribati or "democratic people’s republic of korea" or "republic of korea" or north korea or south korea or korea or kosovo or kyrgyzstan or kirghizia or kirgizstan or "kyrgyz republic" or kirghiz or laos or "lao pdr" or "lao people's democratic republic" or lebanon or "lebanese republic" or lesotho or basutoland or liberia or libya or "libyan arab jamahiriya" or madagascar or "malagasy republic" or malawi or nyasaland or malaysia or "malay federation" or "malaya federation" or maldives or "indian ocean islands" or "indian ocean" or mali or micronesia or "federated states of micronesia" or "marshall islands" or nauru or "northern mariana islands" or palau or tuvalu or mauritania or mauritius or mexico or moldova or moldovian or mongolia or montenegro or morocco or ifni or mozambique or "portuguese east africa" or myanmar or burma or namibia or nepal or "netherlands antilles" or nicaragua or niger or nigeria or "republic of north macedonia" or macedonia or north macedonia or pakistan or panama or "papua new guinea" or paraguay or peru or philippines or philipines or phillipines or phillippines or russia or "russian federation" or ussr or "soviet union" or "union of soviet socialist republics" or rwanda or ruanda or samoa or "pacific islands" or polynesia or "samoan islands" or "navigator island" or "navigator islands" or "sao tome and principe" or senegal or serbia or "sierra leone" or melanesia or "solomon island" or "solomon islands" or "norfolk island" or "norfolk islands" or somalia or "south africa" or "south sudan" or "sri lanka" or ceylon or "saint kitts and nevis" or "st. kitts and nevis" or "saint lucia" or "st. lucia" or "saint vincent and the grenadines" or "saint vincent" or "st. vincent" or grenadines or sudan or suriname or surinam or "dutch guiana" or "netherlands guiana" or syria or "syrian arab republic" or tajikistan or tadjikistan or tadzhikistan or tadzhik or tanzania or tanganyika or thailand or siam or "timor leste" or "east timor" or togo or "togolese republic" or tonga or tunisia or turkey or turkmenistan or turkmen or uganda or ukraine or uzbekistan or uzbek or vanuatu or "new hebrides" or venezuela or vietnam or "viet nam" or "middle east" or "west bank" or gaza or palestine or yemen or zambia or zimbabwe or "northern rhodesia" or "global south" or "africa south of the sahara" or "sub saharan africa" or "subsaharan africa" or "africa, central" or "central africa" or "africa, northern" or "north africa" or "northern africa" or magreb or maghrib or sahara or "africa, southern" or "southern africa" or "africa, eastern" or "east africa" or "eastern africa" or "africa, western" or "west africa" or "western africa" or "west indies" or "indian ocean islands" or caribbean or "central america" or "latin america" or "south and central america" or "south america" or "asia, central" or "central asia" or "asia, northern" or "north asia" or "northern asia" or "asia, southeastern" or "southeastern asia" or "south eastern asia" or "southeast asia" or "south east asia" or "asia, western" or "western asia" or "europe, eastern" or "east europe" or "eastern europe" or "developing country" or "developing countries" or "developing nation" or "developing nations" or "developing population" or "developing populations" or "developing world" or "less developed country" or "less developed countries" or "less developed nation" or "less developed nations" or "less developed population" or "less developed populations" or "less developed world" or "lesser developed country" or "lesser developed countries" or "lesser developed nation" or "lesser developed nations" or "lesser developed population" or "lesser developed populations" or "lesser developed world" or "under developed country" or "under developed countries" or "under developed nation" or "under developed nations" or "under developed population" or "under developed populations" or "under developed world" or "underdeveloped country" or "underdeveloped countries" or "underdeveloped nation" or "underdeveloped nations" or "underdeveloped population" or "underdeveloped populations" or "underdeveloped world" or "middle income country" or "middle income countries" or "middle income nation" or "middle income nations" or "middle income population" or "middle income populations" or "low income country" or "low income countries" or "low income nation" or "low income nations" or "low income population" or "low income populations" or "lower income country" or "lower income countries" or "lower income nation" or "lower income nations" or "lower income population" or "lower income populations" or "underserved country" or "underserved countries" or "underserved nation" or "underserved nations" or "underserved population" or "underserved populations" or "underserved world" or "under served country" or "under served countries" or "under served nation" or "under served nations" or "under served population" or "under served populations" or "under served world" or "deprived country" or "deprived countries" or "deprived nation" or "deprived nations" or "deprived population" or "deprived populations" or "deprived world" or "poor country" or "poor countries" or "poor nation" or "poor nations" or "poor population" or "poor populations" or "poor world" or "poorer country" or "poorer countries" or "poorer nation" or "poorer nations" or "poorer population" or "poorer populations" or "poorer world" or "developing economy" or "developing economies" or "less developed economy" or "less developed economies" or "lesser developed economy" or "lesser developed economies" or "under developed economy" or "under developed economies" or "underdeveloped economy" or "underdeveloped economies" or "middle income economy" or "middle income economies" or "low income economy" or "low income economies" or "lower income economy" or "lower income economies" or "low gdp" or "low gnp" or "low gross domestic" or "low gross national" or "lower gdp" or "lower gnp" or "lower gross domestic" or "lower gross national" or lmic or lmics or "third world" or "lami country" or "lami countries" or "transitional country" or "transitional countries" or "emerging economies" or "emerging nation" or "emerging nations") | Cochrane LMIC Filter - Central |
| Web of Science | CU=(afghanistan or albania or algeria or "american samoa" or angola or argentina or armenia or armenian or azerbaijan or bangladesh or "republic of belarus" or belarus or byelarus or belorussia or byelorussian or belize or "british honduras" or benin or dahomey or bhutan or bolivia or "bosnia and herzegovina" or bosnia or herzegovina or botswana or bechuanaland or brazil or brasil or bulgaria or "burkina faso" or "burkina fasso" or "upper volta" or burundi or urundi or "cabo verde" or "cape verde" or cambodia or kampuchea or "khmer republic" or cameroon or cameron or cameroun or "central african republic" or "ubangi shari" or chad or china or colombia or comoros or "comoro islands" or "iles comores" or mayotte or "democratic republic of the congo" or "democratic republic congo" or congo or zaire or "costa rica" or "cote d’ivoire" or "cote d’ ivoire" or "cote divoire" or "cote d ivoire" or "ivory coast" or cuba or djibouti or "french somaliland" or dominica or "dominican republic" or ecuador or egypt or "united arab republic" or "el salvador" or "equatorial guinea" or "spanish guinea" or eritrea or eswatini or swaziland or ethiopia or fiji or gabon or "gabonese republic" or gambia or "georgia (republic)" or georgia or georgian or ghana or "gold coast" or grenada or guatemala or guinea or "guinea bissau" or guyana or "british guiana" or haiti or hispaniola or honduras or india or indonesia or timor or iran or iraq or "isle of man" or jamaica or jordan or kazakhstan or kazakh or kenya or kiribati or "democratic people’s republic of korea" or "republic of korea" or north korea or south korea or korea or kosovo or kyrgyzstan or kirghizia or kirgizstan or "kyrgyz republic" or kirghiz or laos or "lao pdr" or "lao people's democratic republic" or lebanon or "lebanese republic" or lesotho or basutoland or liberia or libya or "libyan arab jamahiriya" or madagascar or "malagasy republic" or malawi or nyasaland or malaysia or "malay federation" or "malaya federation" or maldives or "indian ocean islands" or "indian ocean" or mali or micronesia or "federated states of micronesia" or "marshall islands" or nauru or "northern mariana islands" or palau or tuvalu or mauritania or mauritius or mexico or moldova or moldovian or mongolia or montenegro or morocco or ifni or mozambique or "portuguese east africa" or myanmar or burma or namibia or nepal or "netherlands antilles" or nicaragua or niger or nigeria or "republic of north macedonia" or macedonia or north macedonia or pakistan or panama or "papua new guinea" or paraguay or peru or philippines or philipines or phillipines or phillippines or russia or "russian federation" or ussr or "soviet union" or "union of soviet socialist republics" or rwanda or ruanda or samoa or "pacific islands" or polynesia or "samoan islands" or "navigator island" or "navigator islands" or "sao tome and principe" or senegal or serbia or "sierra leone" or melanesia or "solomon island" or "solomon islands" or "norfolk island" or "norfolk islands" or somalia or "south africa" or "south sudan" or "sri lanka" or ceylon or "saint kitts and nevis" or "st. kitts and nevis" or "saint lucia" or "st. lucia" or "saint vincent and the grenadines" or "saint vincent" or "st. vincent" or grenadines or sudan or suriname or surinam or "dutch guiana" or "netherlands guiana" or syria or "syrian arab republic" or tajikistan or tadjikistan or tadzhikistan or tadzhik or tanzania or tanganyika or thailand or siam or "timor leste" or "east timor" or togo or "togolese republic" or tonga or tunisia or turkey or turkmenistan or turkmen or uganda or ukraine or uzbekistan or uzbek or vanuatu or "new hebrides" or venezuela or vietnam or "viet nam" or "middle east" or "west bank" or gaza or palestine or yemen or zambia or zimbabwe or "northern rhodesia" or "global south" or "africa south of the sahara" or "sub saharan africa" or "subsaharan africa" or "africa, central" or "central africa" or "africa, northern" or "north africa" or "northern africa" or magreb or maghrib or sahara or "africa, southern" or "southern africa" or "africa, eastern" or "east africa" or "eastern africa" or "africa, western" or "west africa" or "western africa" or "west indies" or "indian ocean islands" or caribbean or "central america" or "latin america" or "south and central america" or "south america" or "asia, central" or "central asia" or "asia, northern" or "north asia" or "northern asia" or "asia, southeastern" or "southeastern asia" or "south eastern asia" or "southeast asia" or "south east asia" or "asia, western" or "western asia" or "europe, eastern" or "east europe" or "eastern europe" or "developing country" or "developing countries" or "developing nation" or "developing nations" or "developing population" or "developing populations" or "developing world" or "less developed country" or "less developed countries" or "less developed nation" or "less developed nations" or "less developed population" or "less developed populations" or "less developed world" or "lesser developed country" or "lesser developed countries" or "lesser developed nation" or "lesser developed nations" or "lesser developed population" or "lesser developed populations" or "lesser developed world" or "under developed country" or "under developed countries" or "under developed nation" or "under developed nations" or "under developed population" or "under developed populations" or "under developed world" or "underdeveloped country" or "underdeveloped countries" or "underdeveloped nation" or "underdeveloped nations" or "underdeveloped population" or "underdeveloped populations" or "underdeveloped world" or "middle income country" or "middle income countries" or "middle income nation" or "middle income nations" or "middle income population" or "middle income populations" or "low income country" or "low income countries" or "low income nation" or "low income nations" or "low income population" or "low income populations" or "lower income country" or "lower income countries" or "lower income nation" or "lower income nations" or "lower income population" or "lower income populations" or "underserved country" or "underserved countries" or "underserved nation" or "underserved nations" or "underserved population" or "underserved populations" or "underserved world" or "under served country" or "under served countries" or "under served nation" or "under served nations" or "under served population" or "under served populations" or "under served world" or "deprived country" or "deprived countries" or "deprived nation" or "deprived nations" or "deprived population" or "deprived populations" or "deprived world" or "poor country" or "poor countries" or "poor nation" or "poor nations" or "poor population" or "poor populations" or "poor world" or "poorer country" or "poorer countries" or "poorer nation" or "poorer nations" or "poorer population" or "poorer populations" or "poorer world" or "developing economy" or "developing economies" or "less developed economy" or "less developed economies" or "lesser developed economy" or "lesser developed economies" or "under developed economy" or "under developed economies" or "underdeveloped economy" or "underdeveloped economies" or "middle income economy" or "middle income economies" or "low income economy" or "low income economies" or "lower income economy" or "lower income economies" or "low gdp" or "low gnp" or "low gross domestic" or "low gross national" or "lower gdp" or "lower gnp" or "lower gross domestic" or "lower gross national" or lmic or lmics or "third world" or "lami country" or "lami countries" or "transitional country" or "transitional countries" or "emerging economies" or "emerging nation" or "emerging nations") | Cochrane LMIC Filter - Central |
| Google Scholar | “mHealth”, “postpartum”, “family planning”, “LMICs” |  |
| National Grey Literature Conference | mobile health postpartum family planning |  |
| National Grey Literature Conference | mobile health postnatal contraceptive |  |
| National Grey Literature Conference | mHealth postpartum family planning |  |
| MEDLINE (Qualitative only) | ((phone adj3 call*) or ((cell* or mobile or smart or google or nexus or iphone) adj3 (phone* or telephone*)) or smartphone* or smart-phone* or (blackberr* not extract) or (black-berr* not extract) or (mobile adj3 health) or mhealth or m-health or e-health* or ehealth* or (electronic* adj1 health) or (mobile adj3 technol*) or ((mobile or smartphone or smart-phone or phone or software) adj3 app*) or MMS or multimedia messaging service or SMS or short messag* service or (text* adj3 messag*) or text-messa* or voice messag* or interactive voice response or IVR or (social adj3 media or network*) or telemedicine or tele-medicine or telehealth or tele-health or telecare or tele-care or mobile app*).mp. | Smith et al. 2015  Allida et al. 2012  Goncalves-Bradley et al. 2012 |
| MEDLINE (Qualitative only) | exp Telemedicine/ or exp Cell Phone/ or exp Text Messaging/ or exp Internet/ or exp Mobile Applications/ | Smith et al. 2015  Allida et al. 2012  Goncalves-Bradley et al. 2012 |
| MEDLINE (Qualitative only) | ((condom* or (OC pill) or (depot medroxyprogest* or NET-EN or NET EN or Mesigyna or Cyclofem) or (intrauterine system or intra- uterine system or IUS or intrauterine device* or intra-uterine device* or IUD*) or (vasectomy or sterilisation or sterilization or (tubal ligation)) or ((vaginal ring) or cycletel or cycle-tel or ((abstain or abstinen*) adj2 (sex* or intercourse)) or lactational amenorr*)) or (contracept* or family planning or (birth adj (control or regulat* or spacing)) or planned parenthood or ((population* or fertility) adj3 (regulat* or control))) or (pregnan* adj2 (adolescen* or teen* or schoolchild*)) or (pregnan* adj3 (prevent* or interrupt* or unplanned or unwanted or mistimed))).mp. | Smith et al. 2015 |
| MEDLINE (Qualitative only) | exp Family Planning Services/ or exp Contraception/ or exp Reproductive Behavior/ or exp Contraception Behavior/ or exp Contraceptive agents/ or exp Contraceptive Devices/ or exp Pregnancy in Adolescence/ or exp Pregnancy, Unplanned/ or exp Pregnancy, Unwanted/ or exp Abortion, Induced/ | Smith et al. 2015 |
| MEDLINE (Qualitative only) | ((contracept* or (vasectomy or sterilisation or sterilization) or (pregnan* or abortion)) or (NORPLANT or implanon or Femplant)).mp. | Smith et al. 2015 |
| MEDLINE (Qualitative only) | (postpartum or post-partum or postnatal or post-natal).mp. |  |
| MEDLINE (Qualitative only) | exp Postpartum Period/ |  |
| MEDLINE (Qualitative only) | (afghanistan or albania or algeria or "american samoa" or angola or argentina or armenia or armenian or azerbaijan or bangladesh or "republic of belarus" or belarus or byelarus or belorussia or byelorussian or belize or "british honduras" or benin or dahomey or bhutan or bolivia or "bosnia and herzegovina" or bosnia or herzegovina or botswana or bechuanaland or brazil or brasil or bulgaria or "burkina faso" or "burkina fasso" or "upper volta" or burundi or urundi or "cabo verde" or "cape verde" or cambodia or kampuchea or "khmer republic" or cameroon or cameron or cameroun or "central african republic" or "ubangi shari" or chad or china or colombia or comoros or "comoro islands" or "iles comores" or mayotte or "democratic republic of the congo" or "democratic republic congo" or congo or zaire or "costa rica" or "cote d’ivoire" or "cote d’ ivoire" or "cote divoire" or "cote d ivoire" or "ivory coast" or cuba or djibouti or "french somaliland" or dominica or "dominican republic" or ecuador or egypt or "united arab republic" or "el salvador" or "equatorial guinea" or "spanish guinea" or eritrea or eswatini or swaziland or ethiopia or fiji or gabon or "gabonese republic" or gambia or "georgia (republic)" or georgia or georgian or ghana or "gold coast" or grenada or guatemala or guinea or "guinea bissau" or guyana or "british guiana" or haiti or hispaniola or honduras or india or indonesia or timor or iran or iraq or "isle of man" or jamaica or jordan or kazakhstan or kazakh or kenya or kiribati or "democratic people’s republic of korea" or "republic of korea" or north korea or south korea or korea or kosovo or kyrgyzstan or kirghizia or kirgizstan or "kyrgyz republic" or kirghiz or laos or "lao pdr" or "lao people's democratic republic" or lebanon or "lebanese republic" or lesotho or basutoland or liberia or libya or "libyan arab jamahiriya" or madagascar or "malagasy republic" or malawi or nyasaland or malaysia or "malay federation" or "malaya federation" or maldives or "indian ocean islands" or "indian ocean" or mali or micronesia or "federated states of micronesia" or "marshall islands" or nauru or "northern mariana islands" or palau or tuvalu or mauritania or mauritius or mexico or moldova or moldovian or mongolia or montenegro or morocco or ifni or mozambique or "portuguese east africa" or myanmar or burma or namibia or nepal or "netherlands antilles" or nicaragua or niger or nigeria or "republic of north macedonia" or macedonia or north macedonia or pakistan or panama or "papua new guinea" or paraguay or peru or philippines or philipines or phillipines or phillippines or russia or "russian federation" or ussr or "soviet union" or "union of soviet socialist republics" or rwanda or ruanda or samoa or "pacific islands" or polynesia or "samoan islands" or "navigator island" or "navigator islands" or "sao tome and principe" or senegal or serbia or "sierra leone" or melanesia or "solomon island" or "solomon islands" or "norfolk island" or "norfolk islands" or somalia or "south africa" or "south sudan" or "sri lanka" or ceylon or "saint kitts and nevis" or "st. kitts and nevis" or "saint lucia" or "st. lucia" or "saint vincent and the grenadines" or "saint vincent" or "st. vincent" or grenadines or sudan or suriname or surinam or "dutch guiana" or "netherlands guiana" or syria or "syrian arab republic" or tajikistan or tadjikistan or tadzhikistan or tadzhik or tanzania or tanganyika or thailand or siam or "timor leste" or "east timor" or togo or "togolese republic" or tonga or tunisia or turkey or turkmenistan or turkmen or uganda or ukraine or uzbekistan or uzbek or vanuatu or "new hebrides" or venezuela or vietnam or "viet nam" or "middle east" or "west bank" or gaza or palestine or yemen or zambia or zimbabwe or "northern rhodesia" or "global south" or "africa south of the sahara" or "sub saharan africa" or "subsaharan africa" or "africa, central" or "central africa" or "africa, northern" or "north africa" or "northern africa" or magreb or maghrib or sahara or "africa, southern" or "southern africa" or "africa, eastern" or "east africa" or "eastern africa" or "africa, western" or "west africa" or "western africa" or "west indies" or "indian ocean islands" or caribbean or "central america" or "latin america" or "south and central america" or "south america" or "asia, central" or "central asia" or "asia, northern" or "north asia" or "northern asia" or "asia, southeastern" or "southeastern asia" or "south eastern asia" or "southeast asia" or "south east asia" or "asia, western" or "western asia" or "europe, eastern" or "east europe" or "eastern europe" or "developing country" or "developing countries" or "developing nation" or "developing nations" or "developing population" or "developing populations" or "developing world" or "less developed country" or "less developed countries" or "less developed nation" or "less developed nations" or "less developed population" or "less developed populations" or "less developed world" or "lesser developed country" or "lesser developed countries" or "lesser developed nation" or "lesser developed nations" or "lesser developed population" or "lesser developed populations" or "lesser developed world" or "under developed country" or "under developed countries" or "under developed nation" or "under developed nations" or "under developed population" or "under developed populations" or "under developed world" or "underdeveloped country" or "underdeveloped countries" or "underdeveloped nation" or "underdeveloped nations" or "underdeveloped population" or "underdeveloped populations" or "underdeveloped world" or "middle income country" or "middle income countries" or "middle income nation" or "middle income nations" or "middle income population" or "middle income populations" or "low income country" or "low income countries" or "low income nation" or "low income nations" or "low income population" or "low income populations" or "lower income country" or "lower income countries" or "lower income nation" or "lower income nations" or "lower income population" or "lower income populations" or "underserved country" or "underserved countries" or "underserved nation" or "underserved nations" or "underserved population" or "underserved populations" or "underserved world" or "under served country" or "under served countries" or "under served nation" or "under served nations" or "under served population" or "under served populations" or "under served world" or "deprived country" or "deprived countries" or "deprived nation" or "deprived nations" or "deprived population" or "deprived populations" or "deprived world" or "poor country" or "poor countries" or "poor nation" or "poor nations" or "poor population" or "poor populations" or "poor world" or "poorer country" or "poorer countries" or "poorer nation" or "poorer nations" or "poorer population" or "poorer populations" or "poorer world" or "developing economy" or "developing economies" or "less developed economy" or "less developed economies" or "lesser developed economy" or "lesser developed economies" or "under developed economy" or "under developed economies" or "underdeveloped economy" or "underdeveloped economies" or "middle income economy" or "middle income economies" or "low income economy" or "low income economies" or "lower income economy" or "lower income economies" or "low gdp" or "low gnp" or "low gross domestic" or "low gross national" or "lower gdp" or "lower gnp" or "lower gross domestic" or "lower gross national" or lmic or lmics or "third world" or "lami country" or "lami countries" or "transitional country" or "transitional countries" or "emerging economies" or "emerging nation" or "emerging nations").mp. | Cochrane LMIC Filter - Central |
