# Supplementary material for: mHealth interventions for postpartum family planning in LMICs: A realist review
Source: PLOS Glob Public Health. 2024 Jul 18;4(7):e0003432. doi: 10.1371/journal.pgph.0003432 (PMC11257288; doi:10.1371/journal.pgph.0003432)
Supplement: S1 Data — (DOCX) [file pgph.0003432.s004.docx]

##
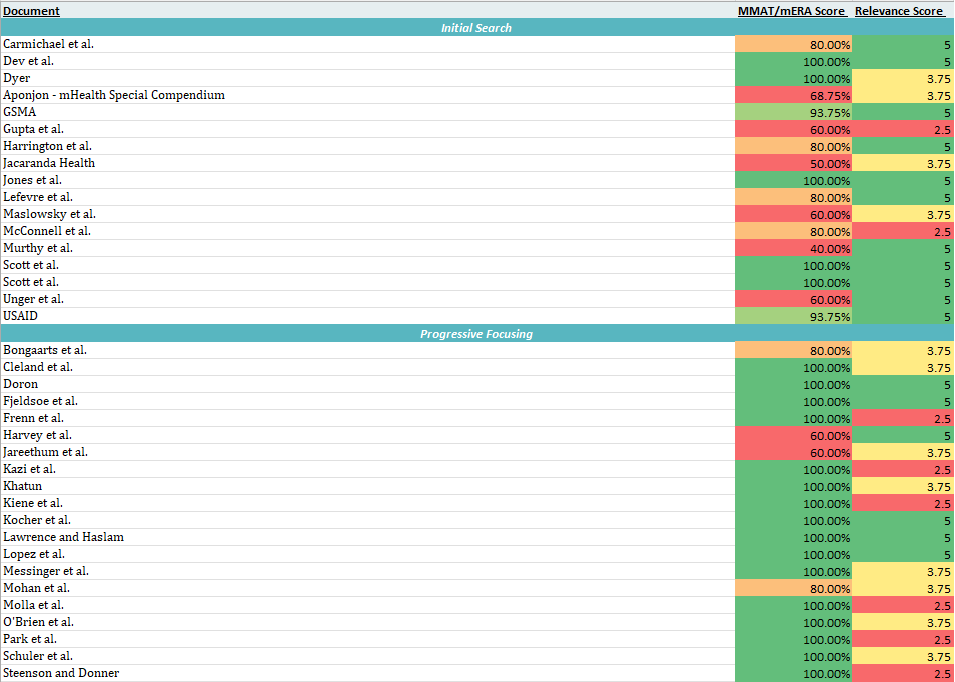
Appendix D – Quality Appraisal

Figure IV. Heat map of rigour and relevance scores for included documents.

Detailed relevance and rigour assessments can be found here ([Realist Review Data Extraction Appraisal V4 7-23](https://lshtm-my.sharepoint.com/personal/lsh2101218_lshtm_ac_uk/Documents/PhD%20Materials/Realist%20Review/Realist%20Review%20Data%20Extraction&Appraisal%20V4%207-23.xlsx?web=1)).

**MMAT Score Legend**

0-70% = Low methodological rigour, 71-80% = Moderate methodological rigour, 80-100% = High methodological rigour

% Score = $\frac{No. of MMAT or mERA criteria met}{Total number of Eligible criteria} \times100$

**Relevance Score Legend**

0-3 = Low relevance, 3-4 = Moderate relevance, 5 = High relevance

Relevance scores were determined by AC through multiple readings of included texts and consideration of each text’s contributions to CMOC building in this realist review.
