# Supplementary material for: mHealth interventions for postpartum family planning in LMICs: A realist review
Source: PLOS Glob Public Health. 2024 Jul 18;4(7):e0003432. doi: 10.1371/journal.pgph.0003432 (PMC11257288; doi:10.1371/journal.pgph.0003432)
Supplement: Attachment — Submitted filename: Comments for PLOS Global Public Health Peer Review.docx [file pgph.0003432.s007.docx]

mHealth interventions for postpartum family planning in LMICs: A Realist Review

Reviewer Comments:

This is an important work given the heterogeneity of the contexts in which mHealth interventions have been applied. The authors do an excellent job detailing the purpose of a realist review and its importance to answering their research question.

Major Comments:

Abstract:

Updating your inclusion criteria seems potentially biasing, particularly given your point on “using pre-determined inclusion criteria” as an aspect of a realist review. It could help to explain why this was done in the methods section.

This manuscript focuses primarily on engagement with mHealth programs, which makes sense. However, the ultimate question is whether this engagement leads to subsequent behavior change regarding PPFP. It would be helpful to mention this briefly in the abstract and make it a greater point of emphasis throughout the manuscript.

Introduction:

“postpartum family planning interventions addressing these contexts are most likely to result in improvements in infant and child mortality rates” – Does this ‘most likely’ mean they are better at improving mortality rates than other programs or that these are the health outcomes they are best suited to improve?

Define unmet need for modern contraception– is this simply not having access to contraception during the 24-month interval at which these greater risks are present or just a general lack of access to modern contraception to prevent unwanted pregnancy?

There are two distinct points being made regarding lack of access to modern contraception in the opening paragraph. The first would be a lack of the actual materials for modern contraception (i.e., condoms, birth control, etc.). The second is a lack of access to contraceptive counseling (i.e. information on preventing unwanted pregnancy). It appears this manuscript focuses more directly on the latter. Does the systematic review and meta-analysis cited focus on this lack of information or a lack of ability to procure these actual materials? You’ll need a justification for why addressing the social and behavioral barriers to access should prove effective.

Small typo “their effectiveness *has* been evaluated for using randomized controlled trials”

What is the mixed evidence of mHealth intervention effectiveness in the RMNCH landscape? Are there some reviews suggesting mHealth interventions are ineffective and what outcomes did they look at?

It makes sense to include studies regardless of methodological rigor here given the scope of a realist review. However, did the authors give differential weight to better designed studies?

Materials and Methods:

Initial Programme Theory – How did you decide on this particular program and these documents for extracting the CMOCs? You should provide some justification for what makes them so well suited for this task. It also seems that this program is the ultimate focus of the review, which should be discussed earlier in the introduction given how central it is to the manuscript. This isn’t currently clear in the introduction or abstract.

Why were only 10% of full texts double-screened? Typically in systematic reviews, the full text studies are also screened by both screeners

In the PICOS table, should be clear on what these “PPFP outcomes” are – what counts as a PPFP outcome and what does not? What PPFP outcomes can mHealth interventions realistically improve?

How did you determine the search terms to use for the progressive focusing stage of the review?

Results:

These results focus primarily on the degree to which women engage with the programs, which is undoubtedly important but what about this gained knowledge/awareness affecting actual contraceptive use? It would be helpful to link this all back to the most important question, which is the degree to which context affects uptake of PPFP after engagement with these programs.

“However, there was still some evidence that automated voice-calls were potentially more effective at delivering mHealth programmes to women with low digital literacy compared to SMS messages” – What is this evidence exactly? The rest of the paragraph suggests that these automated voice-calls are ineffective with this demographic and the following paragraph seems to contradict this

Discussion:

In the first sentence of the discussion, it seems more accurate to say that mHealth interventions are more effective when these barriers to phone access, use, and ownership do not exist. It’s not clear how these interventions can target such barriers.

You should at least mention in the results when the studies being discussed are those that “lack methodological rigor.” It seems unlikely we’d want to draw equally strong inferences from such studies compared to those that are better conducted.

In the discussion, it would also be helpful to outline what made these studies low in methodological rigor. This could help the reader understand how strongly to consider such evidence.
