# Supplementary material for: mHealth interventions for postpartum family planning in LMICs: A realist review
Source: PLOS Glob Public Health. 2024 Jul 18;4(7):e0003432. doi: 10.1371/journal.pgph.0003432 (PMC11257288; doi:10.1371/journal.pgph.0003432)
Supplement: Attachment — Submitted filename: Response to reviewers - RR Pub 6-24 vFinal.docx [file pgph.0003432.s008.docx]

Abinaya Chandrasekar
The London School of Hygiene & Tropical Medicine
Keppel Street, London, WC1E 7HT
[abinaya.chandrasekar@lshtm.ac.uk](mailto:abinaya.chandrasekar@lshtm.ac.uk)

6/11/2024

Dr. Catherine Kyobutungi and Prof. Madhukar Pai
PLOS Global Public Health

Dear Editors,

We appreciate the time and effort that you and the reviewers have dedicated to evaluating our manuscript titled “mHealth interventions for postpartum family planning in LMICs: A realist review”, submitted for consideration in PLOS Global Public Health. We have carefully considered the comments provided by the reviewers and have revised our manuscript to address these concerns. Below, we provide a point-by-point response to each of the reviewers' comments.

Abstract:

1. Updating your inclusion criteria seems potentially biasing, particularly given your point on “using pre-determined inclusion criteria” as an aspect of a realist review. It could help to explain why this was done in the methods section.

- Response: Thank you for your comment. The following sentence on page 5 of the Introduction section outlines where the pre-determined inclusion criteria is useful, specifically only to initially narrow the scope of the realist review.

*“Using a pre-determined inclusion criteria and a systematic search strategy is beneficial to initially narrow the scope of a realist review (31,32)”.*

We have included an additional sentence in the Methods section on page 7 to further emphasize this point.

*“To initially narrow the scope of the review, we used a systematic search strategy and pre-determined inclusion criteria.”*

We hope this provides clarification to the reader that using a pre-determined inclusion criteria is beneficial for the initial stages of a realist review and not for the progressive focusing stages carried out later in the realist review.

2. This manuscript focuses primarily on engagement with mHealth programs, which makes sense. However, the ultimate question is whether this engagement leads to subsequent behaviour change regarding PPFP. It would be helpful to mention this briefly in the abstract and make it a greater point of emphasis throughout the manuscript.

- Response: We agree that it would be beneficial to signpost to the reader more clearly that this manuscript details the impact of contexts and conditions on mHealth programme engagement. We have added clarification in the abstract on page 3 to address this comment:

*“Although the impact of mHealth programmes on postpartum family planning outcomes have been systematically reviewed, the contexts, conditions, and mechanisms underpinning programme engagement and their* *impact on outcomes remain unclear.”*

Introduction:

3. “postpartum family planning interventions addressing these contexts are most likely to result in improvements in infant and child mortality rates” – Does this ‘most likely’ mean they are better at improving mortality rates than other programmes or that these are the health outcomes they are best suited to improve?

- Response: What we are trying to say is that postpartum family planning interventions are more successful at improving infant and child mortality rates when they address the adverse consequences of short birth intervals. We have made the following amendment in the Introduction section on page 4 to address this comment:

*“postpartum family planning (PPFP) interventions addressing these contexts are more likely to result in improvements in infant and child mortality rates when they address the adverse consequences of short birth intervals (4).”*

4. Define unmet need for modern contraception – is this simply not having access to contraception during the 24-month interval at which these greater risks are present or just a general lack of access to modern contraception to prevent unwanted pregnancy?

- Response: We have added a sentence in the Introduction section on page 4 to provide a clear definition for the unmet need for family planning:

*“The unmet need for family planning is the percentage of all married women who want to delay or limit future pregnancies but are not using any form of contraception (5).”*

5. There are two distinct points being made regarding lack of access to modern contraception in the opening paragraph. The first would be a lack of the actual materials for modern contraception (i.e., condoms, birth control, etc.). The second is a lack of access to contraceptive counseling (i.e. information on preventing unwanted pregnancy). It appears this manuscript focuses more directly on the latter. Does the systematic review and meta-analysis cited focus on this lack of information or a lack of ability to procure these actual materials? You’ll need a justification for why addressing the social and behavioral barriers to access should prove effective.

- Response: We have added two additional sentences on page 4 of the Introduction section to address this comment:

*“Although the unmet need for family planning is also driven by sociocultural barriers such as gender norms, limited supply of contraceptives, and poor integration of family planning counselling into existing health services, the distal factor of PPFP knowledge is still a significant predictor of contraceptives uptake. This review focuses on programmes that aim to elicit PPFP behaviour change through improvements in knowledge and awareness of PPFP methods.”*

6. Small typo “their effectiveness *has* been evaluated for using randomized controlled trials”

- Response: Thank you for flagging this. We have made the suggested change on page 4 in the Introduction section.

7. What is the mixed evidence of mHealth intervention effectiveness in the RMNCH landscape? Are there some reviews suggesting mHealth interventions are ineffective and what outcomes did they look at?

- Response: We have added additional information to address this comment, as we feel it would be helpful for the reader to understand the breadth of the evidence being cited to illustrate our point. We added the following sentences to the Introduction section on page 5 to address this comment:

*“Several systematic reviews also report mixed evidence of the effectiveness of mHealth interventions in the RMNCH landscape on uptake and adherence of contraception and the prevention of unintended pregnancies (19-23). Smith et al. and Zulu et al. report a positive association between text-messaging interventions and oral contraceptive uptake across mostly high-income country (HIC) settings (19-20). Mangone et al.’s systematic review and content analysis of mobile applications concluded that several interventions were ineffective at reducing unintended pregnancies due to the promotion of contraceptive methods with low effectiveness (22).”*

8. It makes sense to include studies regardless of methodological rigor here given the scope of a realist review. However, did the authors give differential weight to better designed studies?

- Response: We did not differentially weight studies with better designs. To help address this issue, we have added the following concluding sentence on page 5 at the end of the Introduction section:

*“In realist reviews, studies are included on the basis of relevance to theory building; transparency of rigour of included documents is still a key reporting criterion (34) to contextualise findings from the reviewed evidence.”*

We also included the following sentence on page 9 of the Materials and Methods section to provide further clarification:

*“We did not differentially weight studies for inclusion in this review on the basis of methodological rigour.”*

9. There is scope to make the introduction crisper by tightening it.

- Response: We have removed a few sentences from the Introduction section (page 5 and page 6) to make this section more concise.

Materials and Methods

10. Initial Programme Theory – How did you decide on this particular program and these documents for extracting the CMOCs? You should provide some justification for what makes them so well suited for this task. It also seems that this program is the ultimate focus of the review, which should be discussed earlier in the introduction given how central it is to the manuscript. This isn’t currently clear in the introduction or abstract.

- Response: We have added the following clarification on page 6 of the Materials and Methods section to illustrate our decision-making process during the development of the IPT.

*“The MAMA series of interventions, of which mMitra is a part, was an appropriate starting point for scoping literature to develop the IPT because of the scope of this project, in which the realist review detailed in this manuscript forms the basis of a realist evaluation of the mMitra programme. The review and evaluation components were designed to inform each other.”*

Further clarification on the importance of the mMitra programme and its association with this realist review is provided in an additional paragraph at the end of the Introduction section on page 6. We have attached further details on the mMitra programme in an additional appendix (Appendix A).

*“This realist review occurred in conjunction with and was informed by a realist evaluation of an automated voice-messaging programme based in India (‘mMitra’) which aims to improve socioeconomically vulnerable women’s access to pregnancy and postpartum care information. Further details on the design and implementation of the mMitra programme is provided in Appendix A.”*

11. Why were only 10% of full texts double-screened? Typically in systematic reviews, the full text studies are also screened by both screeners.

- Response: Thank you for your comment. We considered double-screening 10% of full texts to be sufficient since realist reviews involve an embedded procedure of iterative and theory driven re-searching of the literature. Also, double-screening 100% of the titles and abstracts ensures that we sufficiently verified that the inclusion criteria were applied correctly. These processes mitigate the risk of missing relevant articles for inclusion in the study. To clarify this point and address this comment, we have added the following amendment to pages 7-8 of the Material and Methods section:

*“Double-screening a random 10% sample of full texts is common practice in realist reviews (41,42). Realist reviews are a form of configurational review. If any documents with relevant data were to be missed, it is unlikely to introduce significant threats to the validity of the findings. When combined with double-screening 100% of titles and abstracts, these processes ensure that the inclusion criteria were verified and applied suitably.”*

We also increased the reporting transparency of our full text double screening process to address this comment. On pages 9-10 of the Material and Methods section, we added a sentence to clarify the number of discrepancies in full text inclusion decisions between screeners.

*“There were two discrepant screening decisions identified during double-screening of the random 10% sample of full-texts.”*

12. In the PICOS table, should be clear on what these “PPFP outcomes” are – what counts as a PPFP outcome and what does not? What PPFP outcomes can mHealth interventions realistically improve?

- Response: We have added the below clarification to Table I on page 8 to address this comment. We hope this will make it clear to the reader the types of PPFP outcomes we considered relevant for inclusion in this review.

*“Interventions that address PPFP outcomes (e.g. knowledge of PPFP methods, PPFP uptake, adherence to PPFP methods, and prevalence of unintended pregnancies).”*

13. How did you determine the search terms to use for the progressive focusing stage of the review?

- Response: We used a sample of search terms from the initial database search and mechanism-specific terms that would be relevant to the realist review. We framed the terms using Boolean operatives to ensure usefulness on Google Scholar. We have clarified this with the following addendum on page 8 of the Materials and Methods section:

*“These search terms were identified from Cochrane systematic reviews in this topic area and framed using Boolean operatives to ensure usefulness on Google Scholar (19, 43-45).”*

14. Clarify inclusion criteria in the methods section.

- Response: We have added an in-text description of the inclusion criteria used in the systematic search stage of this review. The addendum is presented on page 7 of the Materials and Methods section under the Search and Screening sub-section.

*“Interventions exclusively developed for and delivered by mobile phones were included. Only interventions targeting postpartum family planning outcomes in women within one year postpartum were included. All study designs and methodologies were eligible for inclusion but studies with insufficient detail required for CMOC construction and theory building were excluded from the review.”*

Results

15. These results focus primarily on the degree to which women engage with the programmes, which is undoubtedly important but what about this gained knowledge/awareness affecting actual contraceptive use? It would be helpful to link this all back to the most important question, which is the degree to which context affects uptake of PPFP after engagement with these programmes.

- Response: Thank you for your comment. The focus of this manuscript is the degree to which context affects mHealth programme engagement. The first four sentences of the ‘Barriers of mHealth programme engagement’ sub-section on page 27 state that PPFP uptake is a distal outcome of the engagement outcomes discussed in this paper and that the findings of this review should be interpreted in this way. To further clarify this, we have included an additional sentence on page 27 in the Results section to describe the importance of PPFP knowledge/awareness in the causal path towards behaviour change.

*“It is important to acknowledge that knowledge or awareness are key domains in health behaviour change models, and can potentially impact PPFP behaviour change, although not the sole determinant of outcomes associated with PPFP uptake (82,83).”*

16. “However, there was still some evidence that automated voice-calls were potentially more effective at delivering mHealth programmes to women with low digital literacy compared to SMS messages” – What is this evidence exactly? The rest of the paragraph suggests that these automated voice-calls are ineffective with this demographic and the following paragraph seems to contradict this.

- Response: We have made the amendments specified below on page 27 of the Results section to illustrate the point that some studies continue to report positive associations with voice-messages because they target the issue of digital literacy (e.g. women with low digital literacy are still more effectively captured through voice-messaging programmes compared to text-messaging programmes).

*“However, some studies still reported the benefits of using voice-messaging to deliver health information over text messages because they are more usable by low-literacy populations, while also acknowledging the role of domestic duties in impeding this access. Murthy et al. suggest that voice-messages are a more accessible format for low-literacy populations compared to text messages as a means of potentially improving PPFP knowledge and use (56). Bangladesh’s MAMA-informed programme, Aponjon, found that rural populations and women of low socio-economic status had an unmet need for voice calls to receive health information and potentially yield improved PPFP use due to functional and digital literacy concerns (36).”*

17. Please add sub-headings to clearly state challenges/barriers and facilitators to uptake of the technology.

- Response: We have added titles to mechanisms in each of the CMOCs in Tables III and IV (pages 25 and 29 respectively) to identify whether they outline barriers or facilitators. We have also added sub-headings in the ‘Phone access, use, and ownership’ and ‘Motivation’ sections to separate main findings into ‘Barriers of mHealth programme engagement’ and ‘Facilitators of mHealth programme engagement’ across pages 27-34.

Discussion

18. In the first sentence of the discussion, it seems more accurate to say that mHealth interventions are more effective when these barriers to phone access, use, and ownership do not exist. It’s not clear how these interventions can target such barriers.

- Response: We agree with this comment and have made the following amendments to the Discussion section on page 34:

*“The empirical evidence reviewed in this study suggests that mHealth interventions are potentially more effective when barriers to phone access, use, and ownership are absent and women’s motivation for health-seeking behaviours is encouraged.”*

19. You should at least mention in the results when the studies being discussed are those that “lack methodological rigour”. It seems unlikely we’d want to draw equally strong inferences from such studies compared to those that are better conducted.

- Response: Our response to this comment is an additional sentence added to the Results section, particularly the Quality appraisal sub-section on page 25.

*“Low methodological rigour was attributed to some included grey literature, such as programme documents, because of a lack of transparency in the reporting of fidelity measures, cost assessments, and the replicability of reported findings (49,52). In the academic literature, studies that included inadequate randomization, poor adherence to the assigned intervention, inappropriate qualitative methods to address the research questions, and completeness of the outcome data were considered as having low methodological rigour (51, 59, 65, 66).”*

20. In the discussion, it would also be helpful to outline what made these studies low in methodological rigour. This could help the reader understand how strongly to consider such evidence.

- Response: We agree with this comment, and we hope the following addendum on page 37 in the Discussion section, will provide the reader with the necessary context to determine the rigour of the studies from which some of our findings were sourced.

*“Unblinded randomization processes and poor adherence to the assigned intervention in the Murthy et al. study, for instance, makes it difficult to conclude with certainty whether or not the mMitra programme was effective in improving the adoption of family planning methods.”*

21. In the discussion, please outline what made some studies low in methodological rigour and suggestions for what future studies can incorporate to avoid making the same mistakes. This will also help the readers understand how strongly to consider such evidence.

- Response: Thank you for your comment. We have made explicit the methodological rigour of studies from which we have drawn our conclusions. These changes to the manuscript are addressed in our responses to comments 19 and 20. We have also added an amendment to the Discussion section on page 37 to address this issue.

*“Future studies could improve rigour by increasing transparency of reporting and taking greater steps to protect against bias in exposure and outcome measurement, randomization, and adherence to the interventions.”*

22. Situate your findings in the discussion section by comparing your findings to other similar reviews.

- Response: We agree that this is an important element of any manuscript’s discussion section and have made the following amendments to the Discussion sections on pages 34-36 to address this comment.

*Page 34 – “This realist review addresses the need for identifying and evaluating the components leading to intended and unintended consequences of mHealth interventions and aimed to explain how and why the interventions potentially lead to PPFP outcomes, thus building on the limitations outline in both the Zulu et al. and Smith et al. systematic reviews.”*

*Page 35 – “A systematic review by Aung et al. on the effectiveness of mHealth interventions on family planning uptake in LMICs similarly found that motivational messaging was a key component driving family planning behaviour change (18).”*

*Page 35 – “Family support is one such complex phenomenon. From the findings of this review, familial involvement can activate women’s feelings of support and empowerment to engage with mHealth programmes. Involvement by husbands and relatives can also gatekeep women’s engagement with mHealth programmes and potentially affect the distal outcome of PPFP uptake. Aung et al.’s systematic review presents contradicting evidence on the role of spousal involvement in improving PPFP uptake (18); specifically citing Harrington et al.’s sub-group analysis findings showing no difference in PPFP uptake based on spousal involvement (15). Future research should identify both positive and negative dimensions of familial involvement in mHealth interventions to determine their effects on PPFP knowledge acquisition and uptake.”*

*Page 36 – “This finding builds on the Zulu et al. systematic review which identified the importance of tailoring intervention messaging to account for gender and social norms in order to be contextually relevant and effective at improving PPFP uptake (20).”*

Thank you once again for the opportunity to revise and resubmit our manuscript. We look forward to hearing from you regarding the next steps in the review process.

Sincerely,

Abinaya Chandrasekar
The London School of Hygiene & Tropical Medicine
[abinaya.chandrasekar@lshtm.ac.uk](mailto:abinaya.chandrasekar@lshtm.ac.uk)
